# Targeting and anchoring the mechanosensitive ion channel Piezo to facilitate its inhibition of axon regeneration

**DOI:** 10.1371/journal.pgen.1011968

**Published:** 2025-12-01

**Authors:** Qin Wang, Leanne Miles, Shuo Wang, Lilly M. Ryll, Harun N. Noristani, Ethan Schauer, Ernest J. Monahan Vargas, Jackson Powell, Sean J. O’Rourke-Ibach, Naiara Akizu, Jill Wildonger, Shuxin Li, Yuanquan Song

**Affiliations:** 1 Raymond G. Perelman Center for Cellular and Molecular Therapeutics, The Children’s Hospital of Philadelphia, Philadelphia, Pennsylvania, United States of America; 2 Department of Pathology and Laboratory Medicine, University of Pennsylvania, Philadelphia, Pennsylvania, United States of America; 3 The Graduate Group in Biochemistry and Molecular Biophysics, University of Pennsylvania, Philadelphia, Pennsylvania, United States of America; 4 Center for Neural Development and Repair, Lewis Katz School of Medicine at Temple University, Philadelphia, Pennsylvania, United States of America; 5 Department of Neural Sciences, Lewis Katz School of Medicine at Temple University, Philadelphia, Pennsylvania, United States of America; 6 The Neuroscience Graduate Group, University of Pennsylvania, Philadelphia, Pennsylvania, United States of America; 7 Pediatrics, University of California, San Diego, La Jolla, California, United States of America; 8 Cell & Developmental Biology, School of Biological Sciences, University of California, San Diego, La Jolla, California, United States of America; Universidad de Valparaiso, CHILE

## Abstract

Mechanical force orchestrates a myriad of cellular events including inhibition of axon regeneration, by locally activating the mechanosensitive ion channel Piezo enriched at the injured axon tip. However, the cellular mechanics underlying Piezo localization and function remains poorly characterized. We show that the RNA repair/splicing enzyme Rtca acts upstream of Piezo to modulate its expression and transport/targeting to the periphery of the soma via the Rab10 GTPase, whose expression also relies on Rtca. Loss or gain of function of Rab10 promotes or impedes *Drosophila* sensory neuron axon regeneration, respectively. Rab10 mediates the cell surface expression of integrin β1 (Itgb1)/mys, which colocalizes and genetically interacts with Piezo, facilitating its anchorage and engagement with the microenvironment, and subsequent activation of mechanotransduction to inhibit regeneration. Importantly, loss of Rtca, Piezo1, Rab10 or Itgb1 promotes CNS axon regeneration after spinal cord injury or optic nerve crush in adult mice, indicating the evolutionary conservation of the machinery.

## Author summary

Regarded as the holy grail in regenerative medicine, achieving axon regeneration and functional recovery after neural injury remains a daunting task. Novel strategies to increase the rate and extent of axon regeneration in both the central and peripheral nervous system are desperately needed to ultimately improve functional outcomes in patients with neurological trauma. The effect of mechanical forces in neuronal growth has long been observed. However, not much was known about the underlying molecular and cellular machinery. Our previous work showed that the mechanosensitive ion channel Piezo functions in fly and mouse neurons to inhibit axon regeneration, by sensing and responding to cellular mechanical forces. However, a key question remained unanswered – what controls the expression, localization and activation of Piezo channels? In this study, by combining injury paradigms in fruit fly and mouse, we uncovered three factors that collectively regulate the expression and localization of Piezo, including the RNA repair/splicing enzyme Rtca, a small Rho GTPase Rab10, and a cell surface receptor β1 integrin. Our work reveals a multi-step molecular and cellular machinery controlling Piezo function, fills the knowledge gap on how the activities of various regeneration inhibitors are coordinated, and generated a network of regeneration brakes with hierarchy.

## Introduction

Neurons have varying capacities to regenerate, and nerves in the mature mammalian central nervous system (CNS) retain lower regenerative potential than those in the peripheral nervous system (PNS) [1,2]. Intrinsic regeneration potential varies across types of neurons, including subtypes of retinal ganglion cells (RGCs) and dorsal root ganglion (DRG) neurons [3,4]. Therefore, it is crucial to identify genetic programs that regulate regeneration to provide effective treatments for patients who live with the debilitating effects of nerve damage, ranging from traumatic brain and spinal cord injuries to blindness. Numerous efforts have been made to characterize essential genes and pathways that either promote or inhibit regeneration in the hopes of identifying key therapeutic targets, but current therapies lead to mild recovery at best [5,6].

Utilizing a *Drosophila melanogaster* neural injury paradigm, we have shown that fly sensory neurons exhibit class-specific regeneration capabilities that power the screening of pro- and anti-regeneration factors [7]. In particular, the RNA repair/splicing pathway has emerged as an inhibitory machinery for axon regeneration. The removal of Rtca, an RNA 3’-terminal phosphate cyclase and the core component of this pathway, improved axon repair in the CNS and PNS, while its overexpression suppressed axon regrowth [8]. Whereas *Xbp1* mRNA splicing and the unfolded protein response (UPR) are the key steps modified by Rtca [8,9], the events regulating axon regrowth downstream of Rtca remain sparsely studied.

The effect of mechanical forces on neuronal growth has long been observed. However, little was known about the underlying molecular and cellular machinery. Our prior work showed that Piezo inhibits axon regeneration via its function as a mechanosensitive ion channel. Upon sensing the cellular mechanical force during regeneration, Piezo triggers calcium influx in the growth cone and activates the CamKII-Nos-Atr pathway in fly and mammalian neurons [10,11]. Mutant Piezo protein with prolonged channel opening suppresses axon regeneration, while another mutant that cannot conduct ions fails to inhibit regrowth [11]. However, the million-dollar question remained unanswered – what controls the expression, localization and activation of Piezo channels? How are Piezo channels mobilized and anchored? Understanding and utilizing the mechanotransduction pathway may offer a new dimension to tackle challenges in neural repair and beyond.

Here, we uncovered genetic and cellular mechanisms underlying Piezo localization and function during regeneration. Upstream, the expression of Piezo is modulated by Rtca, which mediates the expression of both Piezo and Rab10, a small Rho GTPase known to function in endosome transport and recycling [12]. Rtca mediates proper localization of Piezo channels to the periphery of the soma via Rab10, and Rab10 also enables Piezo’s enrichment at the injured axon tip. Loss of Rab10 promotes regeneration while its overexpression impedes axon regeneration in fly sensory neurons. Furthermore, Rab10 mediates the cell surface expression of integrin β1 (Itgb1)/mys, which facilitates Piezo’s anchorage and subsequent activation of mechanotransduction to inhibit axon regeneration. Itgb1/mys loss of function (LoF) drastically enhances fly sensory neuron axon regeneration. Importantly, loss of Rtca, Piezo1, Rab10 or Itgb1 boosts CNS axon regeneration in adult mouse spinal cord injury or optic nerve crush models, indicating evolutionary conservation. Our work thus reveals a multi-step molecular and cellular machinery controlling Piezo function, filling the knowledge gap in the coordination of various regeneration inhibitors, and generates a network of regeneration brakes with hierarchy – the targeting of which provides promising opportunities for therapeutic intervention after nerve damage.

## Results

### Inhibiting Rtca promotes axon regeneration and recovery of locomotor functions after SCI in adult mice

**Fig 1:**
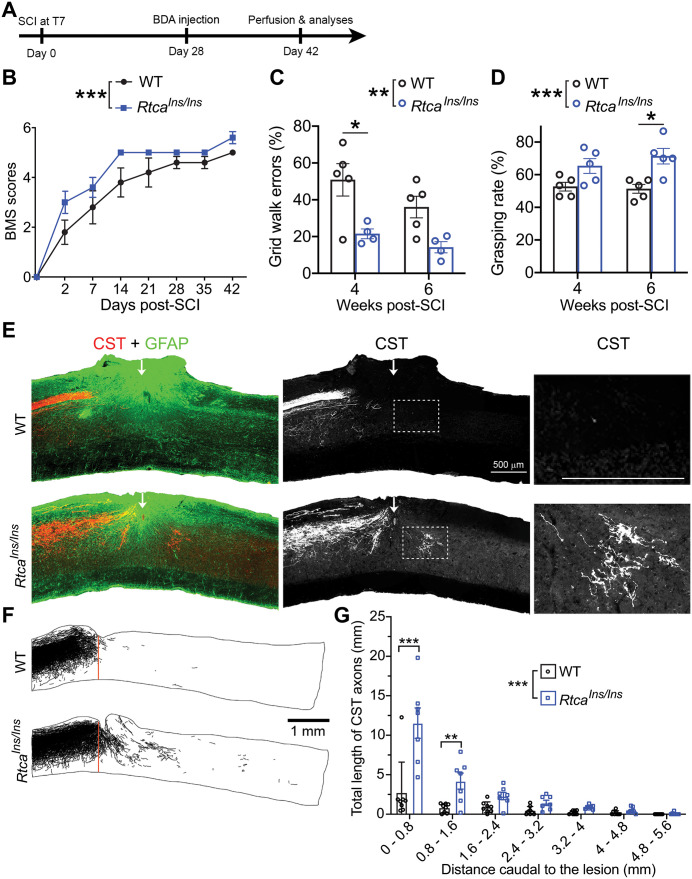
Rtca LoF enhances axon regeneration and recovery of locomotor functions after SCI in mice. (A) Schematic of the experimental protocol for mice SCI. BDA was injected to label CST axons at 28 days after over-hemisection at T7 and mice were perfused at 42 days after injury. (B) The BMS was conducted at multiple time points after SCI. Compared with WT, *Rtca*^*Ins/Ins*^ mice showed better motor improvement and higher BMS scores over six weeks of observation. Two-way ANOVA. *n =* 5 and 5 mice. (C) Grid walk tests were performed at 4- and 6-weeks post SCI. *Rtca*^*Ins/Ins*^ mice made fewer grid-walk errors than the WT mice, especially at 4 weeks after SCI. Two-way ANOVA followed by Sidak’s multiple comparisons test. *n =* 5 and 4 mice. (D) Grasping tests were performed at 4- and 6-weeks post SCI. *Rtca*^*Ins/Ins*^ mice showed significantly increased hindlimb grasping rate, especially at 6 weeks after SCI. Two-way ANOVA followed by Sidak’s multiple comparisons test. *n =* 5 and 5 mice. (E) Representative images showing CST axons and GFAP+ lesion area. Scale bar, 500 μm. (F) Representative examples of camera lucida drawing of CST axons showing that Rtca LoF promotes axon regeneration after SCI. Scale bar, 1 mm. (G) Quantification of total CST axons regrowing beyond the injury site. There are more regrowing axons in *Rtca*^*Ins/Ins*^ mice, especially within the 0-1.6 mm beyond the injury site. Two-way ANOVA followed by Bonferroni’s multiple comparisons test. *n =* 8 and 7 mice. *p < 0.05, **p < 0.01, ***p < 0.001.

Our prior work showed that Rtca LoF not only enhances sensory neuron axon regeneration in the fly CNS, but also modestly promotes RGC axon regeneration after optic nerve crush in adult mice [8]. To further validate Rtca’s conserved role in impeding nerve regeneration in the mammalian CNS, we used the more clinically-relevant spinal cord injury (SCI) paradigm by performing dorsal over-hemisection at thoracic 7 (T7) vertebral level in adult mice (Fig 1A). Since C57BL/6 mice lack the ventral corticospinal tract (CST) axons, our over-hemisection typically transects the majority of spinal cord area and lesions all CST axons [13]. We found that mice with a LoF allele of Rtca – *Rtca*^*Ins/Ins*^ [8] exhibited significantly improved motor recovery as indicated by the increased Basso Mouse Scale (BMS) scores compared with WT mice over the 6 weeks of observation after SCI (Fig 1A and 1B). We used T7 over-hemisection because this model is widely used to study CNS axon regeneration by interrupting defined pathways and sparing bridging tissue for regrowing axons in the ventral spinal cord [14–17]. Several hours after SCI, all mice had similar injury severities, with BMS locomotor scores of 0 (Fig 1B). We observed early partial functional recovery probably due to the sprouting of spared ventral spinal cord axons in both groups of mice, but *Rtca* mutant mice displayed further enhanced locomotor recovery 2–7 days after SCI. A short-range sprouting of some motor axonal tracts, such as serotonergic fibers [18,19], might contribute to the slightly increased BMS scores in *Rtca* mutant mice.

To further assess the locomotor recovery of the hindlimbs after SCI, we also performed the hindlimb grid walk and touch-grasping tests 4 and 6 weeks after SCI. *Rtca*^*Ins/Ins*^ mice significantly surpassed WT in recovery by making fewer grid walk errors and having a higher grasping rate of hindlimbs (Fig 1C and 1D). Several weeks after SCI, *Rtca* mutant mice had a continued increase in BMS scores and showed fewer errors of hindpaw placement and enhanced grasping rate of the hindlimbs. Since it has been reported that contacts of CST axons correlate with functional performance in rodents with SCI [20] and we detected the enhanced CST regrowth in the caudal spinal cord of *Rtca* mutant mice (Fig 1E-1G), CST regeneration might partly contribute to the functional recovery in *Rtca* mutant mice, especially their better coordination of the hindlimbs.

Six weeks after SCI, we evaluated the regeneration of injured CSTs labeled by tracer biotin dextran amine (BDA). CSTs are important for controlling voluntary movements [21], but are particularly refractory to regeneration after axotomy [22–24]. Consistent with the behavioral data, *Rtca*^*Ins/Ins*^ mice showed multiple regenerating CST axons extended beyond the injury site, with the longest axon reaching about 2–3 mm caudal to the lesion (Fig 1E-1G). In contrast, CST axons terminated at the lesion site in WT controls. We carefully checked the CST axons in the caudal spinal cord and confirmed that they meet the previously defined morphological criteria of regenerating axons [25]. Examination of reactive glial scar areas around the lesion site labeled by GFAP indicated that Rtca deletion did not alter glial scar size (S1 Fig). These data demonstrate that Rtca acts as a conserved anti-regeneration factor in the mammalian CNS.

### The RNA repair/splicing pathway regulates axon regeneration

The cellular stress sensor *Xbp1* is known to undergo non-conventional mRNA splicing, mediated by the RNA repair/splicing pathway [26,27]. Properly spliced *Xbp1* mRNA is required for resolving ER stress, as experienced by injured neurons [8]. *Xbp1* splicing requires the ligase RtcB (RNA 2’,3’-cyclic phosphate and 5’-OH ligase), whose enzyme activity is boosted by the catalyst Archease [27]. As a counteraction, Rtca slows down *Xbp1* splicing. Our prior work has demonstrated that LoF of Rtca or Archease promotes or impedes axon regeneration, respectively [8]. The role of RtcB in axon regeneration, however, remains enigmatic. In *C. elegans*, RtcB has been reported to inhibit axon regeneration, independent of the unfolded protein response and Archease [28]. This seeming discrepancy brings up a key question – does RtcB have a conserved role in regeneration or rather function in a species-dependent manner? To address this issue, we generated *RtcB* LoF mutants –*RtcB*^*1-4B*^ by using CRISPR to target a region around 150 bp from the start codon. This results in a 5 bp deletion of the coding sequence, predicted to cause frameshift and protein truncation (S2A Fig). To assess axon regeneration, we used class IV dendritic arborization (C4da) neurons (labeled by *ppk-CD4tdGFP*), which robustly regenerate in wildtype (WT) animals after laser axotomy in the PNS, and found that *RtcB*^*1-4B*^ significantly reduced the percent of regenerating C4da neurons and the regeneration index, which represents normalized axon regeneration length (S2B, S2C, and S2F Fig and Methods). The phenotype is comparable to that of the *Archease* LoF mutant [8], *Archease*^*PBc01013*^ (*Archease*^*PB*^). Moreover, we found that compared with single heterozygotes of *RtcB*^*1-4B*^ or *Archease*^*PB*^, in which C4da neurons exhibit normal regeneration ability, *RtcB* and *Archease* transheterozygotes (*RtcB*^*1-4B*^*/ + ; Archease*^*PB*^*/+*) showed substantially reduced regeneration capacity (S2D and S2E Fig), supporting the hypothesis that RtcB and its cofactor Archease cooperate to allow *Xbp1* splicing and hence axon regeneration (S2A Fig, upper panel). We then wondered if Archease itself is capable of conferring axon regeneration in neurons that normally fail to regrow. We turned to class III dendritic arborization (C3da) neurons (labeled by *19–12-Gal4 > CD4tdGFP; repo-Gal80, nompC-QF* > *mtdTomato* or *nompC-QF* > *mCD8GFP*), which regenerate poorly in WT even after PNS injury. Whereas RtcB overexpression was sufficient to promote axon regrowth, C3da neuron-specific overexpression of Archease was unable to enhance their regeneration (S3A and S3B Fig and Methods), likely because RtcB is the enzyme responsible for the splicing of *Xbp1* mRNA and thus is a limiting factor, while Archease is just the catalyst.

### Rtca maintains proper expression and function of Piezo

**Fig 2:**
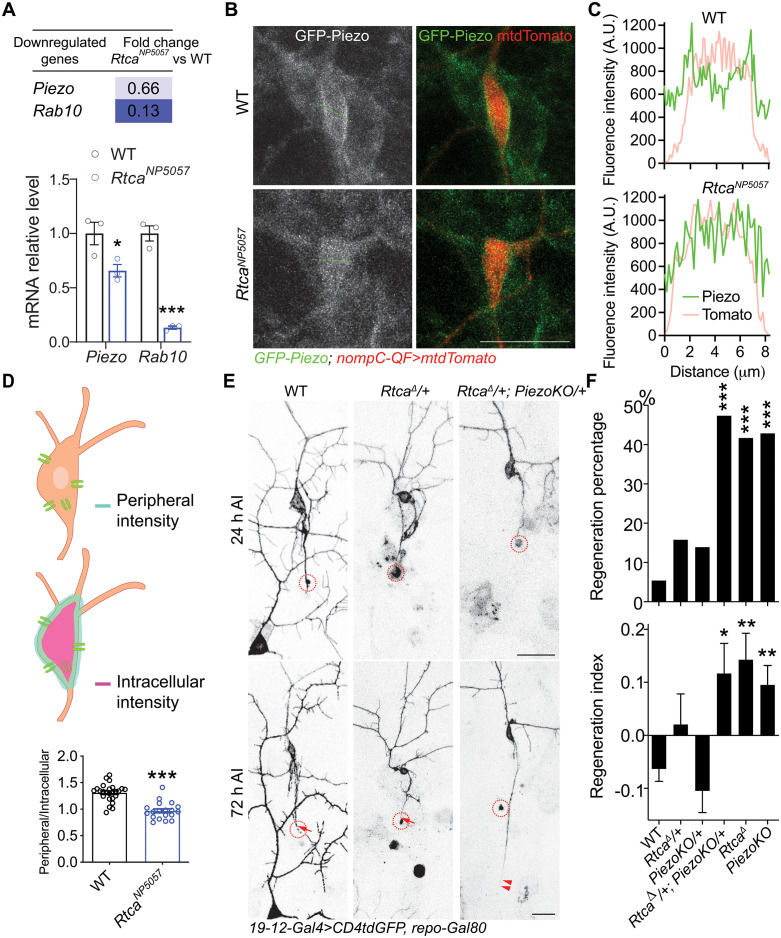
Rtca regulates Piezo’s expression and function. (A) RNA-seq analysis showing both Piezo and Rab10 are downregulated in *Rtca* mutant C4da neurons. The value of *Rtca* mutant is normalized to that of WT. Analyzed by unpaired *t*-test, *n =* 3, *p *= 0.0444, *p* = 2.68 × 10^-4^. (B) Representative images showing that Piezo is enriched on at the periphery of the soma in WT but not in *Rtca* mutants. An example trace is shown in the dotted green line. Scale bar, 20 μm. (C) GFP-Piezo and mtdTomato fluorescence intensity plots reveal that in WT neurons Piezo’s intensity is higher on the at the periphery of the soma than in the intracellular compartment. (D) Quantification of the peripheral/intracellular ratio of GFP-Piezo fluorescence intensity. 3–5 slices (the slice thickness is set as 1-1.2 microns) that encompass the entire cell body are stacked, and the peripheral and intracellular GFP intensity are assessed with ImageJ. For the peripheral intensity, the mean GFP intensity of the entire cell circumference is measured using the line tool. For the intracellular intensity, the mean GFP intensity of the cell soma area within the cell circumference is measured. Unpaired *t*-test with Welch’s correction, *p* < 0.0001. *n =* 25 and 19 neurons. (E and F) Axon regeneration is significantly enhanced in *Rtca* and *Piezo* transheterozygotes. (E) C3da neuron axons were severed and their regeneration was assayed at 72 h AI. The injury site is outlined by the dashed circle, regenerating and non-regenerating axons are marked by arrowheads and arrow. Scale bar, 20 μm. (F) Quantification of axon regeneration shown in (E). Upper panel: regeneration percentage, data are analyzed by Fisher’s exact test, *p* = 0.3237, *p* = 0.2611, *p* = 0.0004, *p* = 0.0008, *p* < 0.0001. Lower panel: regeneration index, analyzed by one-way ANOVA followed by Dunnett’s multiple comparisons test. *n =* 37, 19, 36, 19, 24, and 49 neurons. *p < 0.05, **p < 0.01, ***p < 0.001.

Despite functioning as a potent regeneration inhibitor, how Rtca exerts its function is scarcely studied. To uncover potential Rtca effectors, we previously conducted transcriptome profiling of C4da neurons, comparing WT and *Rtca* LoF mutants – *Rtca*^*NP5057*^ [8]. By focusing on the genes upregulated in *Rtca* mutants, we identified a downstream pro-regeneration factor, ringer, which is suppressed by Rtca [9]. To uncover candidate regeneration inhibitors, we further queried the genes downregulated in *Rtca* mutants and found that *Piezo*’s transcript level is modestly reduced by 34% (Fig 2A). To test whether Piezo’s expression is affected by Rtca at the protein level, GFP was fused to the N-terminal of Piezo to generate the GFP-Piezo fusion protein without disturbing Piezo’s channel function as previously described [11,29]. As a channel protein, Piezo was preferentially enriched on the plasma membrane in WT neuron [30,31]. However, in *Rtca* mutant neurons, Piezo’s peripheral enrichment was largely abolished (Fig 2B and 2C), as indicated by the decreased peripheral/intracellular GFP-Piezo fluorescence intensity ratio (Fig 2D). We tested if the lower intracellular GFP-Piezo intensity is caused by differential expression in the nucleus, and found that in WT GFP-Piezo peripheral/intracellular (with nucleus) ratio and peripheral/cytoplasm (without nucleus) ratio were not significantly different (S4 Fig). To determine whether Rtca and Piezo act in the same genetic pathway, we performed transheterozygotes analysis for knockouts of *Piezo* (*PiezoKO*) and *Rtca* (*Rtca*^*∆*^) [8]. Since both Rtca and Piezo suppress axon regeneration, we turned to C3da neurons which do not robustly regenerate their axons after axotomy. We found that while single heterozygotes of *Rtca*^*∆*^*/+* or *PiezoKO/ +* C3da neurons did not increase regeneration ability, *Rtca* and *Piezo* transheterozygotes (*Rtca*^*∆*^*/ + ; PiezoKO/+*) possessed much stronger regeneration capacity, reaching the level seen in *Rtca* mutants or *PiezoKO* (Fig 2E and 2F), suggesting that Rtca and Piezo genetically interact to suppress axon regeneration and that Piezo likely lies downstream of Rtca.

We next asked whether Rtca regulates Piezo expression via the non-conventional splicing of *Xbp1* mRNA. We found that LoF of Archease, as in the *Archease*^*PB*^ mutant, or C3da neuron-specific overexpression of RtcB failed to significantly alter GFP-Piezo localization to the peripheral cell body (S5A-S5C Fig), suggesting a yet unidentified route of Rtca function in Piezo regulation. We also tested if overexpression of Piezo is capable of rescuing the Rtca LoF phenotype. We found that C3da neuron-specific overexpression of mPiezo1, which suppresses the enhanced C3da neuron axon regeneration seen in *PiezoKO* [11], failed to do so in *Rtca*^*Δ*^ (S5F Fig), likely because mPiezo1 overexpression is incapable of correcting the dysregulated peripheral targeting of Piezo proteins resulting from Rtca LoF.

### Rab10 inhibits axon regeneration downstream of Rtca

**Fig 3:**
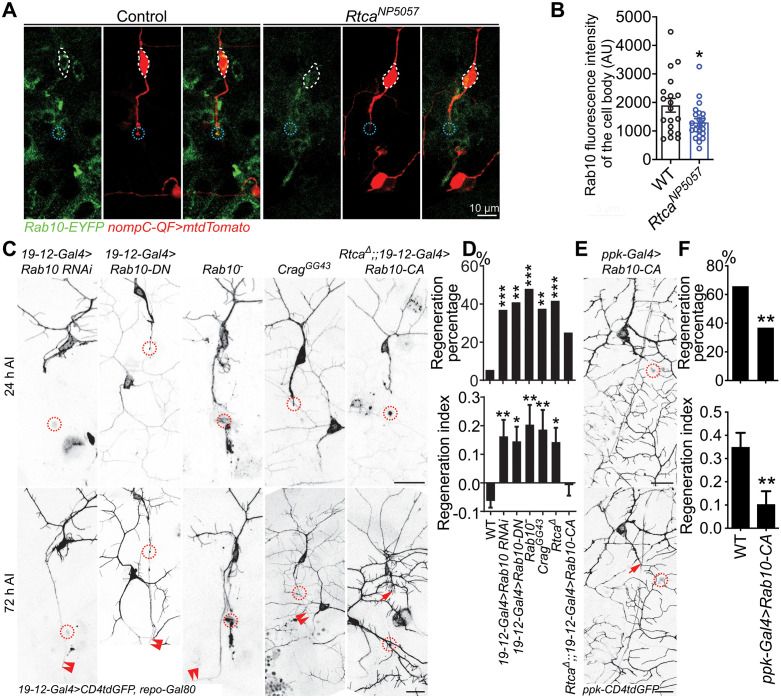
Rab10 operates downstream of Rtca to inhibit regeneration. (A) Rab10 fluorescence intensity is decreased in *Rtca* mutants. C3da cell bodies are marked by a dotted white line, and the injured site by a dotted blue circle. Scale bar, 10 and 5 μm. (B) Quantification of Rab10 fluorescence intensity shown in (A). *n =* 19 and 23 neurons. Analyzed by unpaired *t*-test, *p* = 0.0256. (C and D) Loss of functional Rab10 or inactivating Rab10 promotes axon regeneration, while overexpressing Rab10-CA in *Rtca* mutant attenuates the increased regeneration capacity. (C) C3da neuron axons were severed and their regeneration was assayed at 72 h AI. The injury site is marked by the dashed circle, regenerating and non-regenerating axons are marked by arrowheads and arrow. Scale bar, 20 μm. (D) Quantification of axon regeneration shown in (C). Upper panel: regeneration percentage, data are analyzed by Fisher’s exact test, *p* = 0.0006, *p* = 0.0013, *p* < 0.0001, *p* = 0.0043, *p* = 0.0008, *p* = 0.0837. Lower panel: regeneration index, analyzed by one-way ANOVA followed by Dunnett’s multiple comparisons test. *n =* 37, 46, 22, 33, 24, 24, 20 neurons. (E and F) Overexpressing Rab10-CA decreases regeneration capacity in regeneration-competent C4da neurons. (E) C4da neuron axons were injured and regeneration was assessed at 48 h AI. The injury site is marked by the dashed circle and non-regenerating axon is labeled by arrow. Scale bar, 20 μm. (F) Quantification of axon regeneration by regeneration percentage (Fisher’s exact test, *p* = 0.0267) and regeneration index (unpaired *t*-test with Welch’s correction, *p *= 0.0060). *n =* 37 and 28 neurons. *p < 0.05, **p < 0.01, ***p < 0.001.

Another gene revealed by RNA-seq is Rab10, a member of the diverse Rab family of small GTPases, whose transcription was decreased by 87% in *Rtca* mutant fly neurons (Fig 2A). To validate the decrease of Rab10 in *Rtca* mutants, we utilized a Rab10-EYFP knock-in fly strain [32] to label the endogenous Rab10 and found Rab10 fluorescence intensity was significantly decreased in the cell bodies of injured C3da neurons in *Rtca*^*NP5057*^ mutants (Fig 3A and 3B). We also saw Rab10-EYFP signal present at the axon tip of injured control neurons. To determine if Rab10 inhibits axon regeneration, we first knocked down Rab10 by RNAi in C3da neurons and found these neurons showed enhanced regeneration capacity (Fig 3C and 3D). Similarly, when we overexpressed Rab10^T23N^, a dominant-negative form of Rab10 (Rab10-DN) in C3da neurons, the regeneration percentage and regeneration index were also significantly increased (Fig 3C and 3D). Moreover, *Rab10* knockout (*Rab10*^*-*^) enhanced axon regrowth as well (Fig 3C and 3D). Next, we assessed axon regeneration in *Crag* LoF mutant (*Crag*^*GG43*^) neurons, as Crag operates as a Rab10 guanine nucleotide exchange factor (GEF) responsible for Rab10’s activation [33], and the regeneration capacity consistently increased (Fig 3C and 3D). This evidence collectively demonstrates that inactivating Rab10 promotes axon regeneration. In line with these data, we observed a decrease in regeneration capacity in C4da neurons overexpressing Rab10^Q68L^, a constitutively active form of Rab10 (Rab10-CA), compared to WT C4da neurons, which exhibited robust regeneration after axotomy (Fig 3E and 3F). Lastly, expressing Rab10-CA in *Rtca* mutant C3da neurons attenuated their enhanced regeneration ability (Fig 3C and 3D). While overexpressing Rtca in C4da neurons impaired regeneration, knocking down Rab10 in combination reversed the regeneration reduction (S6A and S6B Fig). Together, these data revealed that Rab10 functions downstream of Rtca to suppress axon regeneration.

### Rab10 mediates Piezo targeting to the peripheral cell body

**Fig 4:**
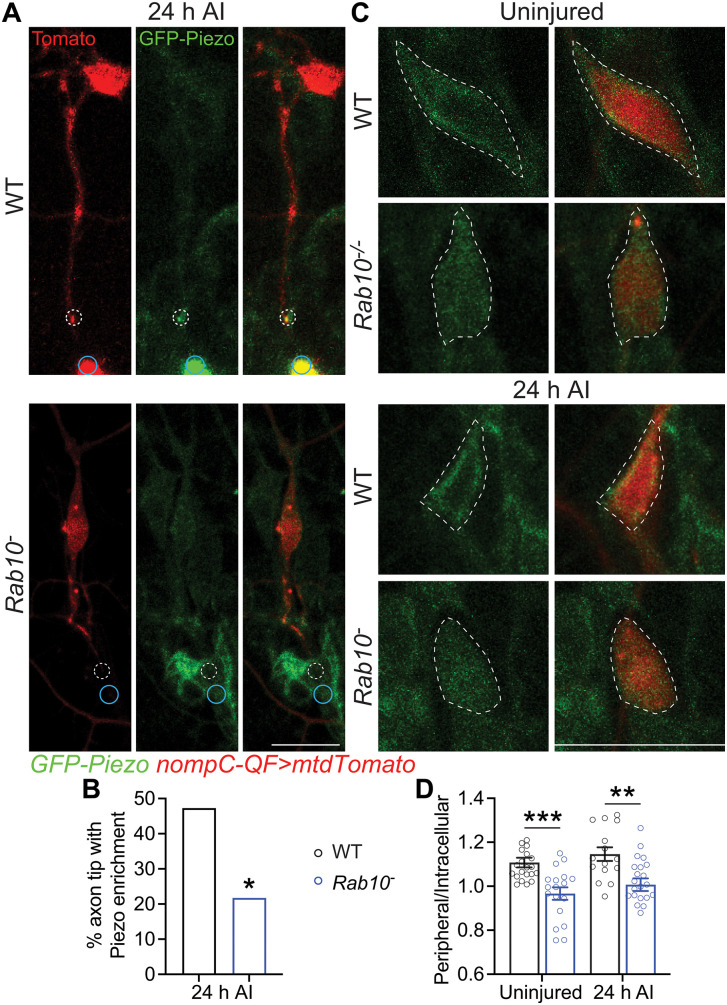
Rab10 mediates Piezo localization. (A) Representative images showing Piezo’s localization in injured neurons. The injury site is marked by blue circle and the growth cone is highlighted by the dotted white circle. Scale bar, 20 μm. (B) Quantification of the percentage of neurons with Piezo accumulating in the injured axonal tip. *n =* 55 and 23 neurons. Analyzed by Fisher’s exact test, *p* = 0.0442. (C and D) Loss of Rab10 reduces Piezo’s peripheral localization after injury. (C) Rab10 LoF impairs Piezo’s peripheral enrichment in both uninjured and injured C3da neurons. (D) Quantification of Piezo peripheral/intracellular ratio. Uninjured: *n =* 21, 27 neurons; 24 h AI: *n =* 15, 22 neurons. Analyzed by two-way ANOVA followed by Sidak’s multiple comparisons test. *p < 0.05, **p < 0.01, ***p < 0.001.

Though Piezo is a well-documented regeneration suppressor, enriching at the injured axon tip to impede axon regrowth by triggering Ca^2+^ signaling [11], how Piezo is recruited to and accumulated at the axon tip after injury remains unknown. Interestingly, Rab10 is involved in a wide range of activities such as vesicular transport in the endosomal/exocytosis recycling pathways [34] and regulation of protein membrane insertion [35,36]. Since both Piezo and Rab10’s expression is regulated by Rtca, and they function in the same pathway as Rtca to inhibit regeneration, we asked if Piezo’s expression and localization are also affected by Rab10. We examined Piezo’s subcellular localization in C3da neurons in the *Rab10* knockout, *Rab10*^*-*^. In 47% of WT neurons, Piezo was enriched at the axon tip after injury, consistent with previous report that Piezo is responsible for detecting the stiffness and regulating Ca^2+^ activity at the growth cone during axon growth [37]. In comparison, only 22% of *Rab10*^*-*^ neurons showed Piezo accumulation at the injured axon tip (Fig 4A and 4B), which was significantly lower than WT. Consistently, the axon tip accumulation of phospho-CamKII (pCaMKII), which is activated by Piezo after axotomy and functions downstream of Piezo to impedes regeneration [11], was also impaired in Rab10 knockdown neurons (S7A and S7B Fig). We then looked at Piezo’s expression and localization at the soma and found Piezo’s peripheral enrichment was impaired in *Rab10*^*-*^ neurons without injury and 24 hours after injury (h AI) (Fig 4C and 4D), indicating that Rab10 is important for Piezo’s proper localization and targeting. Importantly, this result was significant independent from how many optical slices of the soma were analyzed (S8 Fig). This conclusion is further substantiated by the result that C3da neuron overexpression of Rab10-CA was sufficient to increase GFP-Piezo intensity in both the soma and axon (S5A, S5D and S5E Fig). This likely underlies Rab10-CA’s ability to suppress the enhanced axon regeneration in Rtca mutants (Fig 3C and 3D).

A prior report showed that loss of Rab10 led to accumulated and swollen Rab5-positive early endosomes in *C. elegans* intestinal cells [12]. To examine whether Rab10 knockdown affects endosomal trafficking in injured neurons in *Drosophila*, we expressed GFP-Rab5 in C3da neurons and assess GFP fluorescence intensity after axon injury. We noticed that, compared with the dose control (*nompC-Gal4 > BFP*), GFP-Rab5 intensity was significantly increased in Rab10 knockdown neurons at 24 h AI, while at 8 h and 48 h AI the intensity was comparable between the two groups (S7C and S7D Fig). Moreover, the GFP-Rab5 positive puncta area was enlarged in Rab10 knockdown neurons (S7E Fig), suggesting Rab10 is implicated in endosome recycling after injury. Notably, Piezo was observed in GFP-Rab5 positive puncta (S7F Fig).

To confirm the localization pattern of Piezo we had seen, we developed a second tool based on split-GFP. Similar to the published work on Ppk1 [38], we tagged endogenous Piezo with three copies of the split-GFP peptide GFP(11), Piezo::GFP(11)x3, between amino acids G1060 and E1061, which are located in an extracellular loop. This position was selected because a previous study had shown that inserting Myc at the corresponding position in mPiezo1 (P1071) did not affect channel function and resulted in a detectable extracellular tag on the protein [39]. We then expressed a secreted version of GFP(1–10) (secGFP(1–10)) in fat cells using DcG-Gal4, which released secGFP(1–10) into the hemolymph of the larval open circulatory system. Thus, Piezo tagged with GFP(11) only fluoresces when it encounters secGFP(1–10) (S9A Fig). In WT, GFP signal was observed to be enriched at the peripheral cell body in C3da (teal) and C1da (pink) neurons (S9B and S9C Fig). LoF of Rtca or Rab10 led to the accumulation of GFP signal inside the soma with reduced peripheral localization (S9B and S9A Fig). It is worth noting that while secGFP(1–10) binds Piezo::GFP(11)x3 externally on the cellular membrane, it may be endocytosed along with Piezo::GFP(11)x3, leading to fluorescent signal intracellularly.

### Rab10 is required for surface expression of integrin β1, which cooperates with Piezo to inhibit axon regeneration

**Fig 5:**
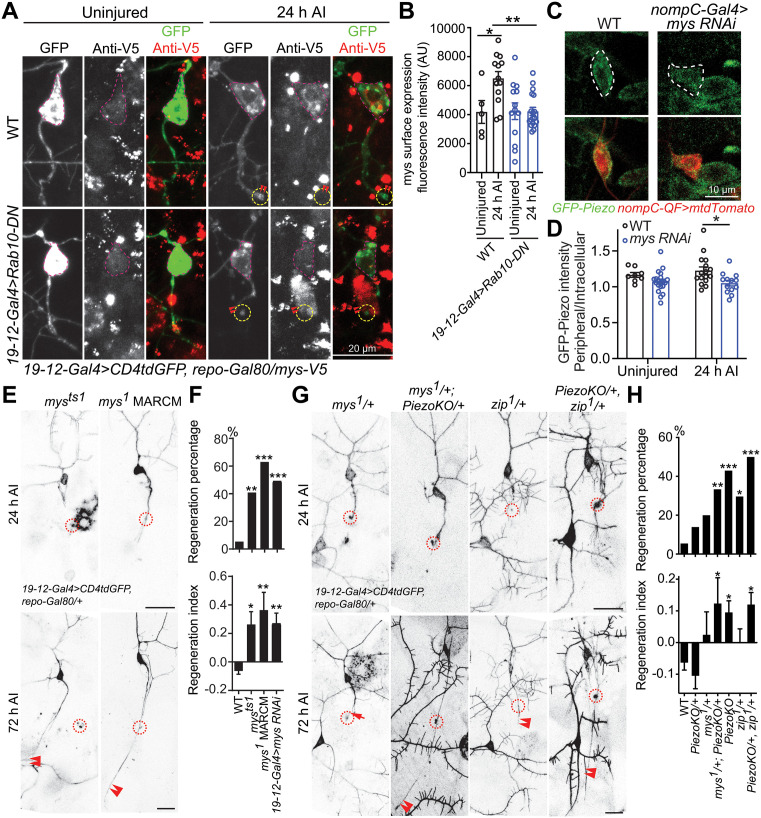
mys genetically interacts with Piezo to suppress axon regrowth. (A) Non-permeabilized immunostaining for V5 is used to assess mys surface expression, which is increased in WT neurons after injury but not in neurons expressing Rab10-DN. C3da cell bodies are marked by magenta dotted lines, and the injury sites are marked by dotted yellow circles. Axon tips are marked by double arrowheads. Scale bar, 20 μm (left panel) and 10 μm (right panel, enlarged view). (B) Quantification of mys surface expression on soma (magenta outline) before and after injury. Somas overlapping with non-specific staining aggregates were NOT analyzed. Analyzed by two-way ANOVA followed by Holm-Sidak’s multiple comparisons test. *n =* 5, 13, 13, and 18 neurons. (C) Knocking down *mys* in C3da neurons reduces Piezo’s peripheral/intracellular GFP-Piezo intensity ratio in C3da neurons after injury. C3da cell bodies are marked by a dotted white line. Scale bar, 10 μm. (D) Quantification of GFP-Piezo fluorescence. Uninjured: *n =* 8, 12 neurons; 24 h AI: *n =* 16 and 13 neurons. Analyzed by two-way ANOVA followed by Holm-Sidak’s multiple comparisons test. (E and F) Axon regeneration is enhanced in *mys* mutants and mys knockdown. C3da neuron axons were severed and their regeneration was assayed at 72 h AI (E). The injury site is marked by the dashed circle and regenerating axon is labeled by arrowheads. Scale bar, 20 μm. Quantification of axon regeneration percentage and index shown in (F). Upper panel: regeneration percentage, data are analyzed by Fisher’s exact test, *p* = 0.0013, *p* < 0.0001, *p* < 0.0001. Lower panel: regeneration index, analyzed by one-way ANOVA followed by Dunnett’s multiple comparisons test. *n =* 37, 22, 16 and 47 neurons. (G and H) Axon regeneration is significantly enhanced in *mys* and *Piezo* transheterozygotes, as well as in *zip* and *Piezo* transheterozygotes. C3da neuron axons were severed and their regeneration was assayed at 72 h AI (G). The injury site is marked by the dashed circle, regenerating and non-regenerating axons are marked by arrowheads and arrow. Scale bar, 20 μm. Quantification of axon regeneration shown in (H). Upper panel: regeneration percentage, data are analyzed by Fisher’s exact test, *p* = 0.2611, *p* = 0.1372, *p* = 0.0044, *p* < 0.0001, *p* = 0.0133, *p* < 0.0001. Lower panel: regeneration index, analyzed by one-way ANOVA followed by Dunnett’s multiple comparisons test. *n =* 37, 36, 15, 33, 49, 27, and 40 neurons. *p < 0.05, **p < 0.01, ***p < 0.001.

We next explored if Rab10 regulates Piezo’s function via additional mechanisms. Prior reports showed that Piezo1’s activity is related to integrin-matrix interactions, and that integrin signaling pathways are essential for Piezo1’s activation upon membrane shear due to fluid flow in endothelial cells [40]. In turn, Piezo1 is important for the activity of Itgb1 in epithelial cells [41]. Moreover, it was previously reported that Rab11 induces Itgb1 surface expression [42]. Considering the synergy and partial redundancy among Rabs in transporting and trafficking [33], we reasoned that Rab10 may play a role in regulating the localization of mys, the fly ortholog of Itgb1, in response to neural injury. We first interrogated the surface expression of mys in C3da neurons using a fosmid transgenic strain that reflects the endogenous expression of mys [43], *mys*^*fTRG00932.sfGFP-TVPTBF*^ (*mys-V5*) and non-permeable immunostaining for the V5 tag. We detected an over 1.5-fold increase of surface mys fluorescence intensity in WT neurons at 24 h AI compared to the uninjured control (Fig 5A and 5B). However, mys surface expression remained unchanged after injury in neurons overexpressing Rab10-DN (Fig 5A and 5B), indicating that the surface expression of mys is increased in response to axotomy in a Rab10-dependent manner. To determine the cellular origin of the mys expression that we had observed, we quantified the anti-mys immunostaining in WT larvae and the anti-V5 surface immunostaining in the *mys-V5* stain, and compared those to C3da neurons with mys knockdown using two independent *mys RNAis*. We found that the immunostaining signal was significantly reduced after mys knockdown with and without axotomy (S10A and S10B Fig), suggesting that at least a substantial fraction of mys comes from C3da neurons. Interestingly, in C3da neurons expressing mys RNAi, Piezo’s peripheral/intracellular GFP-Piezo intensity ratio was modestly decreased after injury (Fig 5C and 5D), potentially implicating mys in regulating Piezo’s anchorage and function. Meanwhile, the percentage of axon tips showing Piezo enrichment at 24 h AI was substantially reduced in mys knockdown neurons compared with WT (S11 Fig).

Piezo’s channel activity is indispensable for its role as a regeneration suppressor [11], therefore, we reasoned that mys, which is important for Piezo’s localization, is involved in the anti-regeneration pathway. We first examined axon regeneration in C3da neuron mys knockdown and two *mys* mutants, *mys*^*ts1*^ (a temperature-sensitive hypomorphic allele [44]) and *mys*^*1*^ (an amorphic allele [44]). Increased C3da neuron axon regrowth was observed in *mys*^*ts1*^ mutants and *mys*^*1*^ MARCM clones as well as mys knockdown (Fig 5E and 5F), confirming that LoF of mys promotes drastic axon regeneration. Then we overexpressed mys along with *Rab10 RNAi* in C3da neurons and found it substantially attenuated the regeneration capacity in Rab10 knockdown neurons, suggesting that mys acts downstream of Rab10 to suppress axon regeneration (S12A and S12B Fig). To determine if Piezo and mys function in the same genetic pathway, we generated transheterozygotes (*mys*^*1*^*/ + ; PiezoKO/+*) and found robust axon regeneration after injury (Fig 5G and 5H). This suggests that mys functions in the same genetic pathway as Piezo to suppress axon regeneration. The transheterozygotes of *Piezo* and *zip*, the fly ortholog of non-muscle myosin II (NM2, an important component in forming cell-matrix adhesions and suppressing regeneration [45]) also displayed strong regeneration capacity (Fig 5G and 5H). This demonstrates that the integrin-matrix interaction is vital for Piezo’s anti-regeneration function. Interestingly, C3da neuron overexpression of mys was unable to suppress the enhanced axon regeneration in Rtca mutants (S5F Fig). This is consistent with the inability of mys overexpression to rescue GFP-Piezo peripheral/intracellular intensity ratio or to increase overall GFP-Piezo soma intensity in *Rtca* mutants (S5G Fig). This data is reminiscent of the mPiezo1 result and contrasts that of Rab10-CA, confirming that mys acts downstream of Rtca and Rab10, constituting only a branch of the signaling.

### Syndapin regulates Piezo enrichment at the injured axon tip and axon regeneration

**Fig 6:**
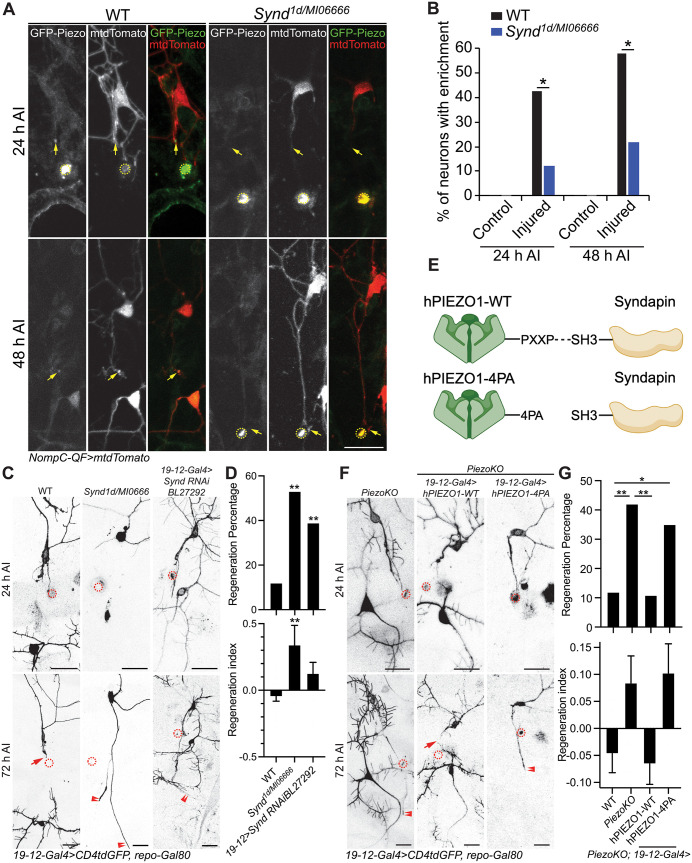
Synd-dependent enrichment of Piezo at the axon tip after injury affects Piezo-induced inhibition of axon regeneration. (A and B) GFP-Piezo is enriched at the axon tip after injury in a Synd-dependent manner. (A) The injury site is marked by the dashed circle and non-regenerating axons are marked by arrows. (B) Quantification of the percentage of neurons with Piezo accumulating in the injured axonal tip. *n* = 8–25 neurons. Two-sided Fischer’s exact tests (percentage) were performed. (C and D) *Synd* LoF in *Synd*^*1d/MI06666*^ transheterozygotes and by C3da neuron knockdown (*Synd RNAiBL27292*) significantly increases regeneration compared to WT. (C) Injury sites demarcated by dashed circle. Regenerating and non-regenerating axons are marked by arrowheads and arrow. Quantification of axon regeneration shown in (D). Upper panel: regeneration percentage, data are analyzed by Fisher’s exact test, *p* = 0.0017, *p =* 0.0079. Lower Panel: regeneration index, analyzed by Kruskal-Wallis test followed by Dunnett’s multiple comparisons test, *p *= 0.0017. *n* = 42, 17 and 31 neurons. (E-G) Protein interaction between hPIEZO1 and Synd is necessary for *PiezoKO* rescue. (E) Mutating the proline-rich PXXP domain of hPIEZO1 (hPIEZO1–4PA) disrupts its potential interaction with Synd. Schematic created in BioRender. Song, Y. (2025) https://BioRender.com/6hb0gzl. (F and G) hPIEZO1 but not hPIEZO1–4PA overexpression in *PiezoKO* C3da neurons significantly reduces regeneration. (F) The injury site is marked by the dashed circle, regenerating and non-regenerating axons are marked by arrowheads and arrow. Quantification of axon regeneration shown in (G). Upper panel: regeneration percentage, data are analyzed by Fisher’s exact test, *p* = 0.0022, *p = *0.0017, *p* = 0.0182. Lower Panel: regeneration index, analyzed by Kruskal-Wallis test followed by Dunnett’s multiple comparisons test. *n* = 42, 50, 24, and 23 neurons. *p < 0.05, **p < 0.005. Scale bar, 20 μm.

To identify additional mechanisms that may promote Piezo enrichment at the injured axon tip, we turned to literature on known Piezo interacting proteins with a role in regulating localization. Previous studies have implicated the Piezo interaction protein PACSIN3 in the subcellular membrane localization of transmembrane channels such as TRPV4 [46] and human PIEZO1 (hPIEZO1) [47]. To test whether Syndapin (Synd), the fly ortholog of PACSIN3, may play a role in the enrichment of Piezo at the axon tip after injury, we assessed Piezo localization in a *Synd* LoF mutant (*Synd*^*1d/MI06666*^, transheterozygotes of *Synd*^*1d*^ [48] and *Synd* ^*MI06666*^ [49]). While Piezo was enriched at the injured axon tip in about 40–60% of WT neurons 24 and 48 h AI, *Synd*^*1d/MI06666*^ neurons demonstrated a significant decrease in enriched axon tips at both time points (Fig 6A and 6B). We next examined the effect of *Synd* LoF mutation and C3da neuron-specific knockdown on axon regeneration and found that both increased regeneration (Fig 6C and 6D) consistent with previous findings where Synd LoF resulted in increased axon length and branching [50]. To determine whether this anti-regenerative effect of Synd was due to its impact on Piezo localization, we generated two transgenic lines which overexpressed either WT hPIEZO1, or a version of hPIEZO1 in which the known proline-rich binding domain (PXXP) responsible for PACSIN3 protein interaction was mutated (hPIEZO1^4PA^) [47] (Fig 6E). While overexpression of hPIEZO1-WT was sufficient to ameliorate the regenerative phenotype of *Piezo* knockout, hPIEZO1^4PA^ overexpression was insufficient (Fig 6F and 6G). Taken together, this data suggests that Piezo-Syndapin protein interaction is important for Piezo enrichment at the injured axon tip, and that this localization of Piezo may be critical in its inhibition of axon regeneration. However, additional experiments that selectively alter Piezo localization to the axon tip would need to be performed to draw any stronger conclusions.

### Rab10, Itgb1 and Piezo1 inhibit axon regeneration in the adult mammalian CNS

**Fig 7:**
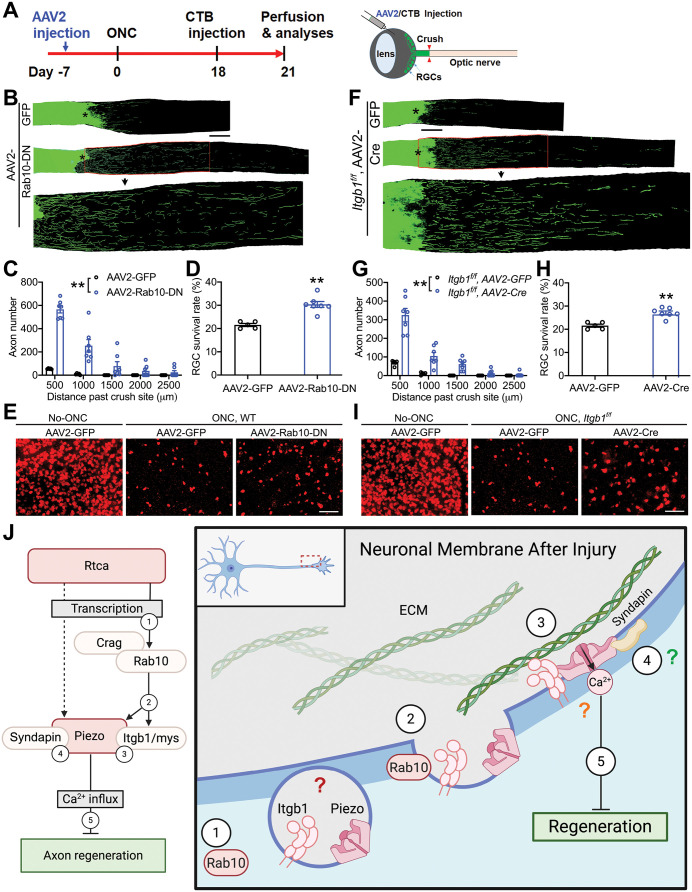
Rab10 and Itgb1 impede CNS nerve regeneration in adult mice. (A) Schematic of experimental protocols for the mice with optic nerve crush. AAV2-GFP, AAV2-Rab10-DN or AAV2-Cre was injected into the retina intravitreally at 7 days before injury. CTB was injected at 18 days after optic nerve crush and mice were perfused at 21 days after injury. (B-E) Loss of functional Rab10 promotes axon regeneration after optic nerve crush. (B) Representative images of optic nerve after crush in control and Rab10-DN overexpression. Scale bar, 100 μm. (C) Quantification of the axon numbers extending beyond the injury cite. (D) Quantification of RGC survival rate after injury. (E) Representative images of the RGCs. *n =* 5 and 7 mice. Scale bar, 50 μm. Data are analyzed by two-way ANOVA (C) or unpaired *t*-test (D). (F-I) *Itgb1* conditional knockout enhances RGC axon regrowth in the mice CNS. (F) Representative images of optic nerve after crush in control and *Itgb1 cKO* mice. Scale bar, 100 μm. (G) Quantification of the axon numbers extending beyond the injury cite. (H) Quantification of RGC survival rate after injury. (I) Representative images of the RGCs. *n =* 4 and 7 mice. Scale bar, 50 μm. Data are analyzed by two-way ANOVA (G) or unpaired *t*-test (H). (J) Schematic drawing depicting how Rtca regulates axon regeneration by mediating Piezo’s localization, created in BioRender. Song, Y. (2025) https://BioRender.com/g44c9nl. It remains to be determined that if Piezo and Itgb1/mys colocalize in Rab10-mediated vesicles (red question mark), if Itgb1/mys physically interact with Piezo to recruit Piezo to the periphery of the soma (orange question mark), and when/where Piezo and Syndapin interact to enrich Piezo in the periphery of the soma (green question mark). **p < 0.01.

Our findings reported above uncovered an anti-regeneration pathway downstream of Rtca. To determine if Rab10 and Itgb1/mys, two critical genes in this pathway, also inhibit regeneration in mammals, we injected AAV2 expressing Rab10-DN into the retina of adult mice and crushed the optic nerves 7 days after AAV2 application (Fig 7A). At 21 days after injury, we observed significantly enhanced axon regeneration in mice treated with AAV2-Rab10-DN but not in the controls treated with AAV2-GFP (Fig 7B and 7C). This result shows that Rab10 impedes CNS axon regeneration in adult rodents. Furthermore, we obtained the *Itgb1*^*f/f*^ mice [51] and deleted the *Itgb1* gene selectively in retinal cells, including RGCs, by intravitreal injection of AAV2 expressing Cre. We found that loss of Itgb1 promoted drastic axon regeneration of axotomized RGCs 21 days after optic nerve crush (Fig 7F and 7G). Interestingly, ablating Itgb1 modestly protected RGCs from injury-induced cell death, which is reminiscent of the phenotype observed in neurons expressing Rab10-DN (Fig 7D, 7E, 7H and 7I). Lastly, we tested RGC-specific conditional knockout of Piezo1 using Cre expression in *Piezo1*^*f/f*^ mice (S13A Fig) similar to Itgb1 above, and found that it modestly promoted RGC survival and axon regeneration (S13B-S13E Fig). Therefore, deleting Rab10, Itgb1 or Piezo1 stimulates significant axon regeneration of injured CNS neurons in adult mammals, and possibly other mechanosensitive ion channels may work in concert with Piezo1 to restrict axon regeneration.

To conclude, we have demonstrated that after injury, Rab10 acts downstream of Rtca to transport Piezo and to upregulate the surface expression of Itgb1/mys, which is important for Piezo’s proper localization to the peripheral cell body/axon tip and inhibition of axon regeneration. In the absence of Rtca, Rab10 is dramatically downregulated. As a result, the axotomy-induced upregulation of surface Itgb1/mys is abolished, accompanied by a decrease in peripheral cell body/axon tip localization of Piezo, perturbing Piezo anchorage and activation, thus facilitating axon regeneration (Fig 7J).

## Discussion

Piezo has been documented as a mechanosensor, able to detect the rigidity and topography of the extracellular matrix in response to the environmental stiffness after axon injury and during regeneration [10]. We have previously shown that Piezo inhibits axon regeneration, and that knocking down Piezo after injury is sufficient to promote axon regeneration [11]. Prior work also demonstrated that Piezo mediates Ca^2+^ influx and triggers downstream pathways in response to injury. Different from L-type Ca^2+^ channels which mediated global calcium transients and promote regeneration [52], the Ca^2+^ influx via the mechanosensitive ion channel exerts an inhibitory effect locally at the axon tips during regenerative timepoints, preventing axon from regrowth [11,53]. But how Piezo is recruited to the injured axon tip remained to be elucidated. The current work revealed an evolutionarily conserved inhibitory pathway for regeneration by regulating Piezo’s localization and function. Based on this study, we posit a model in which Rtca controls the stress-induced mRNA splicing after axotomy. Rab10 functions then downstream of Rtca to increase the surface expression of mys and mediates Piezo’s enrichment and anchorage on the membrane. In parallel, Syndapin functions to modulate Piezo’s subcellular localization via protein-protein interactions, leading to an enrichment at the axon tip after injury. Piezo then senses the mechanical force in the microenvironment, converting it into intracellular signals that prevent axons from regenerating (Fig 7J).

While membrane targeting and anchorage of Piezo by the Rab10-mys pathway seems critical to its enrichment at the injured axon tip, this enrichment process appears to be a more specialized injury response that requires further investigation. It remains to be understood how Syndapin interactions with Piezo lead to post-injury enrichment of Piezo at the axon tip. Additionally, future studies with brighter fluorescent tags and higher resolution imaging are necessary to confirm the described pathway’s control over membranous localization of Piezo. Due to the relatively weak fluorescent intensity of our endogenously tagged GFP-Piezo, we are only able to implicate Rab10 and mys in Piezo’s general localization to the periphery of the cell body and enrichment at the axon tip.

Our findings link the injury-induced RNA repair/splicing to membrane trafficking. During cellular stress, Rtca is recruited to mediate RNA metabolism. Prior results demonstrated that Rtca is an anti-regeneration factor [8]. Here we further demonstrated that in flies, Rtca and RtcB function oppositely in axon regeneration. Whereas RtcB cooperates with Archease to unconventionally splice *Xbp1*, generating an activated *Xbp1* [27], which benefits myelin removal and axon regeneration [54], Rtca antagonizes the reaction to slow down *Xbp1* splicing, impeding axon regrowth [8]. However, many of the downstream effectors in the Rtca pathway during regeneration have not been explored, dependent or independent of Xbp1 splicing. *Rtca* mutant RNA-seq data showed that in fly sensory neurons, the expression levels of more than 200 genes were altered compared to WT. Our prior work showed that the microtubule-associated protein (MAP) ringer/TPPP3 is highly upregulated in *Rtca* mutants, which interacts with futsch/MAP1B and HDAC6 to modulate microtubule dynamics and promotes axon regeneration (S14 Fig) [9]. Interestingly, Gene Ontology (GO) analysis reflected that the pathways regulating extracellular matrix and plasma membrane topped the list of those affected in *Rtca* mutants [9]. Intriguingly, Rab10, a critical regulator of membrane organization and composition by controlling post-Golgi vesicle trafficking and endosomal sorting [34], is among the genes downregulated most strikingly in the absence of Rtca. Piezo is another gene downregulated in *Rtca* mutants, and Rtca LoF was shown to reduce Piezo localization to the periphery of the soma. Therefore, Rtca has multiple signaling branches, with the Piezo pathway outlined in this study being one of them. We found that overexpression of the constitutively active form of Rab10 (Rab10-CA) suppresses the regeneration enhancement in *Rtca* LoF, likely because it is both overexpression and the CA form, resulting in overall increased Piezo expression throughout the cell.

Rab10 has been reported to promote axon and dendrite outgrowth during development via mediating membrane trafficking [35,36,55], which is essential for localizing trans-membrane proteins (*e.g.*, DMA-1 and HPO-30) to the membrane [55,56]. Upon neural injury, several proteins related to endosome sorting and membrane trafficking are upregulated, including Rab4 and rabaptin-5 [57], suggesting that this pathway dynamically regulates injury-induced cellular stress. Here we showed that after axon injury, Rab10 depletion interferes with early endosome recycling, leads to delayed endosome transport and accumulated Rab5^+^ endosomes, which may impair the biological processes depending on the endosome network and in response to axotomy. Piezo is observed in Rab5-labeled early endosomes suggesting that the endocytic recycling pathway may contribute to the increased Piezo’s tip concentration after injury, as an energy-saving strategy adopted by injured neurons. However, future experiments would be required to confirm Piezo trafficking in Rab5-labeled early endosomes. Integrin, whose expression and distribution are mainly controlled by endocytosis and recycling [58], is also severely affected in Rab10 knockdown neurons. Since Rab10 function in endosome transport and recycling, and mys/integrin is essential to the endocytic recycling pathway, this could explain how Rab10, mys and Piezo function through the same genetic pathway to repress axon regeneration. Previously, Rab10 was shown to directly interact with Itgb1 and colocalize with integrins in endosomal versicles [59]. We found that in intact neurons, the surface expression of mys is comparable between WT and Rab10-DN, while after injury mys surface expression increases in WT but not Rab10-DN neurons. This suggests that Rab10 is necessary for mys’ quick response to injury-induced stress, although other Rab members may compensate for Rab10 and properly localize mys to the membrane. Overexpression of mys was unable to rescue proper localization of Piezo in Rtca LoF animals and did not suppress regeneration. These results are consistent with mys being able to act on the Piezo protein, while all other aspects of the pathway are still dysregulated. Overexpression of mys modestly suppressed the regeneration in Rab10 knockdown animals. This is likely because the Rab10 knockdown is only partial, which does not fully disrupt Piezo function and can be compensated by the extra mys protein.

Integrins link the interactions between cells and the surrounding matrix, and are potentially critical for wound healing [60] and axonal growth/guidance [61]. Nevertheless, the role of integrins during axon outgrowth and regrowth is controversial. Although integrins α7 and α9 act as pro-regeneration factors in rodents [62,63], Itgb1 suppresses axon growth in the spinal cord [64]. Moreover, Kingston *et. al.* found that loss of integrin β3 not only protects RGCs from cell death, but also promotes RGC axon regeneration after optic nerve injury [65]. In comparison, our findings showed that deleting mys/Itgb1 drastically enhances axon regeneration in both flies and mice while increasing RGC survival in mice moderately. Given that α and β integrin subunits interact with each other and can form functional heterodimers [66], cells may dynamically adjust the membrane expression of different integrin subunits corresponding to the complex environment to mediate regeneration after injury. However, further studies are needed to determine the precise compositions of integrin subunits for promoting cell survival, axon regeneration, and functional recovery.

Mechanosensation is one of the main functions of integrin-mediated cells and extracellular matrix adhesion, and integrin functions as a mechanosensor itself [67]. Piezo is associated with integrin-mediated mechanotransduction [66]. A prior report has shown that upon force, Piezo1’s recruitment and stabilization in adhesions correlates with the enrichment of β1 integrins while anticorrelates with β3 integrins, suggesting that integrin may bind in a complex with Piezo to regulate Piezo’s recruitment [40]. Here we showed that mys genetically interacts with Piezo to regulate Piezo’s localization and function after injury. Considering that Piezo is observed in Rab5-labeled early endosomes, increasing Piezo’s concentration at the injured axonal tip through the endocytic recycling pathway together with integrins is potentially an energy-saving strategy adopted by injured neurons. However, further studies are required to show whether Piezo and Itgb1/mys colocalize to Rab10-mediated vesicles, and whether they physically interact in the membrane. Notably, Piezo’s expression and localization are altered in the absence of Rtca even before injury, so we are unable to rule out the possibility that Rtca directly regulates Piezo expression. Interestingly, zip/NM2 also functions in the same genetic pathway as Piezo to inhibit axon regeneration, suggesting that cell and extracellular matrix adhesion is necessary for Piezo’s function during regrowth. Our lab previously found that Piezo operates to convert environment stiffness/rigidity into intracellular signaling and activates the Atr-Chek1 pathway to regulate axon regeneration [10]. These collectively suggest that after injury, the Rtca-Rab10-mys pathway facilitates Piezo’s enrichment at the axon tip, where Piezo senses the stiffness of the matrix and triggers anti-regeneration signaling. Furthermore, our results showed that Rab10, mys and Synd are all required for the enrichment of Piezo at the injured axon tip. Future experiments to locally disrupt Piezo axon tip enrichment, for example via optogenetic approaches, are warranted to provide more definitive evidence regarding how important this subcellular localization of Piezo is in its function in axon regeneration. The fact that this anti-regenerative pathway is conserved from *Drosophila* to mice is encouraging and emboldens the use of the fly model to screen antagonists of nerve repair.

## Methods

### Ethics statement

All experiments in this study were performed and carried out according to the protocols approved by the Institutional Animal Care and Use Committee (IACUC) at Temple University (approval IDs: SCI-#5095 and ONC-#5096) and the Institutional Biosafety Committee (IBC) at the University of Pennsylvania (approval IDs: Song 16-000049).

### Fly stocks

*19-12-Gal4* [68]*, repo-Gal80* [69]*, ppk-CD4-tdGFP* [70]*, ppk-Gal4* [70]*, UAS-CD4tdGFP* [70]*, nompC-Gal4* [71], *nompC-QF* [71], *QUAS-mtdTomato* [72], *QUAS-mCD8GFP* [72], GFP-Piezo [11], *Rtca*^*NP5057*^ [8], *Rtca*^*∆*^ [8]*,UAS-Rtca* [8] and *PiezoKO* [11], *UAS-mPiezo1* [11], UAS-*hPIEZO1-WT* [11], *Rab10*^*EYFP*^ [32], *Rab10*^*-*^ [73], *UAS-YFP.Rab10.Q68L* (*Rab10-CA*) [74], *UAS-YFP.Rab10.T23N* (*Rab10-DN*) [74], *mys*^*1*^ [44], *mys*^*ts1*^ [44], *zip*^*1*^ [75], *UAS-secGFP(1–10)* [38], *Dcg-Gal4* [76], *Archease*^*PBc01013*^ [8], *Synd*^*1d*^ [48], *Synd* ^*MI06666*^ [49]) and *UAS-RedStinger* [77] have been previously described. *UAS-Rab10 RNAi* (BL#26289), *UAS-GFP-Rab5* (BL#43336)*, UAS-mys RNAi* (BL#33642), *UAS-mys* (BL#68158), *UAS-mRFP* (BL#7119) and *UAS-BFP* (BL#56807) were acquired from Bloomington stock center, and *UAS-mys RNAi (v103704)* and *mys*^*fTRG00932.sfGFP-TVPTBF*^ (*mys-V5*) (v318285) were acquired from VDRC. Piezo-Flag is a generous gift from Xiang Yang. To generate the *RtcB*^*1-4B*^ alleles, gRNA was cloned into the pU6-3-gRNA vector [78] and injected into Cas9 expressing flies (Rainbow Transgenic Flies, Inc). To generate the *UAS-RtcB* strain, the RtcB cDNA was cloned into the pACU2 vector, then injected to fly embryos (Rainbow Transgenic Flies, Inc) using øC31-based attp40-specific insertion. To generate the UAS-hPIEZO1–4PA strain, the site-directed mutagenesis was performed in the UAS-hPIEZO1-WT plasmid in the pACU2 vector (GenScript), which was then injected to fly embryos (Rainbow Transgenic Flies, Inc) using øC31-based attp2-specific insertion. Randomly selected male and female larvae were used.

*Piezo::GFP(11)x3* was created using CRISPR-Cas9 to target *Piezo*. Three copies of GFP(11), flanked by GGSGG linkers, were inserted into endogenous Piezo between amino acids G1060 and E1061,which are located in an extracellular loop. The edited genomic sequence and resulting protein sequence are below. The gRNA was generated by inserting the targeting sequence (5’-ggatgaaggtcccttcggcg) into the pU6-BbsI-chiRNA plasmid (Addgene #45946) using oligonucleotides (IDT) and KLD enzyme mix reaction (New England Biolabs). The donor template was generated via custom DNA synthesis (Azenta Life Sciences). To generate genome-edited flies, a mixture of the gRNA and donor template was injected into embryos (BestGene Inc.). Sequencing confirmed that GFP(11) was inserted without any additional changes to the genome. Edited genomic sequence (linker is underlined and GFP(11) is bolded; extra nucleotides that were added to preserve the coding frame are in uppercase):

5’-tttccctgggatgaaggtcccttcgGAggtggctctggaggt**agagatcatatggttctccacgaatacgttaacgccgcaggcatcact**ggcggtagtggagga**cgcgaccatatggtactacatgaatatgtcaatgcagccggaataacc**ggagggtccggaggc**cgggatcacatggtgctgcatgagtatgtgaacgcggcgggtataact**ggtgggtcgggcggagGcgagggcatacaacgctgggcgatgctgcca-3’

Protein sequence (linker is underlined and GFP(11) is bolded):

FPWDEGPFGGGSGG**RDHMVLHEYVNAAGIT**GGSGG**RDHMVLHEYVNAAGIT**GGSGG**RDHMVLHEYVNAAGIT**GGSGGGEGIQRWAMLP

### Mice

All studies and procedures involving animal subjects were performed under the approval of the Institutional Animal Care and Use Committee (IACUC) at Temple University. C57BL/6J, *Itgb1*^*f/f*^ and *Piezo1*^*f/f*^ mice were obtained from the Jackson Laboratory. *Rtca*^*Ins/Ins*^ mice was previously described [8]. Adult mice (10 weeks old) with the same sex were randomly assigned to experimental groups. Experiments were performed with both male and female mice, and data was analyzed for sex differences. No sex differences were seen in our data, so all figures reported contain both male and female mice. All mice were housed in an animal facility and maintained in a temperature and light controlled environment with an alternating 12-hour light/dark cycle. The animals had no prior history of drug administration, surgery or behavioral testing.

### Live imaging in flies

Live imaging was performed as described [79]. At the appropriate time, a single larva was anesthetized with ether and mounted on slide with 90% glycerol under coverslips sealed with grease. The larva was then imaged with a Zeiss LSM 880 microscope and returned to grape juice agar plates after imaging sessions. To quantify the localization of Piezo, images were taken by Zeiss LSM880 with the following setting. Width: 136.0308 μm (1024 pixels). Height: 136.0308 μm (1024 pixels). Slice interval: 1-1.2 μm. Resolution: 7.5277 pixels per μm. Voxel size: 0.1328 x 0.1328 x 1 μm^3^. Magnification: 25X oil immersion objective (0.8 NA) with 3X zoom. Confocal pinhole size: 1 unit. Laser power: 488 60%, 594 20%.

### Sensory axon lesion in *Drosophila*

Da sensory neuron injuring and imaging was performed in live fly larvae as described [7,8,80]. Embryos were collected for 2–24 hours on yeasted grape juice agar plates and were incubated at 25 °C. At 48 or 72 h after egg laying, larvae were anesthetized and mounted on glass slide. Up to 7 axons per larvae were severed with a focused 930-nm two-photon laser and the lesions were confirmed 24 h AI. At 48 h AI (for C4da) or 72 h AI (for C3da), axon regeneration was assayed with “regeneration percentage” to quantify the percent of regenerating axons and “regeneration index” to normalize the axon elongation to larvae growth (“axon length”/“distance between the cell body and the axon converging point”) (S2F and S2G Fig), according to published methods [7,8]. An axon was defined as regenerating only when it obviously regenerated beyond the retracted axon stem, and this was independently assessed of regeneration index. When calculating regeneration percentage and index, individual neurons were used as replicates. This is because the variance between individual neurons is similar to the variance across larvae. These neurons are not bundled together and are separable. Each neuron is injured, imaged, and quantified individually. Using values averaged across single larvae would reduce the depth of the data and would be unable to reflect the full spectrum of the variability seen. The regeneration parameters from various genotypes were compared with that of the WT if not noted otherwise, and only those with significant differences were labeled with the asterisks.

### Fly immunohistochemistry and image analysis

Third instar larvae were dissected and their body walls were fixed with 4% PFA according to standard protocols. The tissue was blocked in blocking buffer (PBS + 0.3% Triton X-100 + 5% normal donkey serum) and then incubated with primary antibodies. For mys surface staining, a non-permeabilized immunostaining protocol was used: PBS + 5% normal donkey serum was used for blocking the samples and diluting antibodies. The following primary antibodies were used: rabbit anti-GFP (1:1000, Abcam, ab290), chicken anti-GFP (1:1000, Abcam, ab13970), rabbit anti-RFP (1:1000, Rockland Immunochemicals, 600-401-379), mouse anti-mys (1:1000, DSHB, CF.6G11), mouse anti-V5 (1:400, Thermofisher, MA5–15253) and anti-phospho-CamKII alpha/beta/delta (Thr305) (1:400, Thermofisher, PA5–37832). Fluorescence-conjugated secondary antibodies (1:200, Jackson ImmunoResearch) were used for immunohistochemistry. Larva were mounted in VECTASHIELD Antifade Mounting Medium. To assess Piezo peripheral/intracellular ratio, the boundary of the C3da neuron was defined by membrane-targeting tdTomato (myristoylated and palmitoylated Tomato [72], expressed under the control of *nompC-QF*), then the peripheral and intracellular or cytoplasmic area of the neuron soma were outlined as described in Fig 2D and GFP fluorescence was measured by ImageJ. To image the entire cell body, 9–10 1μm slices were taken. Only 2–5 slices in the middle of the cell body were analyzed to exclude the top and bottom surface of the membrane from our analysis. GFP-Piezo enrichment at the injured axon tip was defined based on the presence of a puncta with an intensity of GFP at least 2x the adjacent axon shaft. Image analyses were performed blind to the genotypes wherever feasible.

### SCI and axon evaluation in adult mice

All studies and procedures involving animal subjects were performed under the approval of the Institutional Animal Care and Use Committee (IACUC) at Temple University. To lesion the spinal cord of WT (controls) and *Rtca*^*Ins/Ins*^ mice (10 weeks old, C57BL/6 background), we exposed the dorsal spinal cord by T6-7 laminectomy. A dorsal over-hemisection (1 mm in depth, and approximately 1.5 mm in dorsoventral diameter) was performed at T7 with a 30-gauge needle and microscissors to completely sever the dorsal spinal cord, including all the CST axons. The lesion depth of 1 mm was ensured by passing a marked 30-gauge needle at least 5 times across the dorsal spinal cord. Four weeks after SCI, the mice received BDA (10 kDa) tracer injections into 5 sites of the sensorimotor cortex (anterior-posterior coordinates from Bregma in mm: 1.0, 0.5, 0, -0.5, -1.0, all at 1.0 mm lateral and at a depth of 1.0 mm). Mice were perfused 2 weeks after BDA injection and fixed spinal cords were dissected for histology.

To compare axon numbers in the caudal spinal cord between WT and *Rtca*^*Ins/Ins*^ groups, we determined the length of BDA-labeled CST axons in all parasagittal sections of the spinal cord from 0 to 4 mm caudal to the lesion epicenter in each animal. The injury center was determined as the midpoint of histological abnormalities produced by lesion cavitation, reactive astrocytes, and morphological changes of injured axons. The CST axons caudal to the lesion were traced manually in each of the parasagittal sections and their total length inside of several bin boxes at 0.8, 1.6, 2.4, 3.2, and 4.0 mm caudal to lesion center was measured with Photoshop and ImageJ software. Tissue sections were immunostained with rat anti-GFAP antibody (1:50, ThermoFisher, 13–0300). We measured the GFAP^+^ dense scar tissue areas and the GFAP^+^ reactive astrocyte areas from multiple parasagittal sections of the lesioned spinal cord in each animal. We defined the former as the areas of densely overlapped GFAP^+^ astrocytic processes around the lesion epicenter and the latter as an obvious increase of GFAP immunoreactivity surrounding the scar tissues.

### Behavioral tests in mice with SCI

To determine functional recovery in *Rtca*^*Ins/Ins*^ mice, we evaluated locomotion alterations during 6 weeks of survival by measuring multiple behavioral tests. The BMS scores were evaluated while the mouse was walking in an open field and confirmed from digital video records. The grid walk errors were counted from videotapes played at a slow speed (4 separate trials per test) and averaged from different trials. The contact-evoked grasping rate was measured by lowering the hindpaws toward a wire cage lid and determining the percent of times that it was grasped successfully. These behavioral tests were performed blindly by two persons who were unaware of animal identifications.

### Optic nerve crush injury, tracer injection, and evaluation of axon regeneration and RGC survival

To access the optic nerve, we used microscissors and dull/angled forceps to gently push away tissues near the eye and avoided damaging the orbital venous sinus. Fine angled forceps were employed to crush the optic nerve for 10 seconds with a consistent pressure at ~1 mm behind the optic disc. To preserve the retinal blood supply, we were careful not to damage the underlying ophthalmic artery. To upregulate Rab10-DN in retina, we injected AAV2-GFP or AAV2-Rab10-DN (2 x 10^12^ genomic copy/mL) intravitreally in adult WT mice and crushed the optic nerves 7 days after AAV2 injection. To delete Itgb1 in the retina of adult *Itgb1*^*f/f*^ mice or Piezo1 in the *Piezo1*^*f/f*^ mice, 7 days before optic nerve crush, we injected AAV2-GFP or AAV2-Cre (2 x 10^12^ genomic copy/ml) intravitreally at the crush side. At 18 days after injury, we labeled regenerating axons by anterogradely injecting Alexa488-conjugated CTB tracer (2 μL per mouse, 2.5 μg/μL) into vitreous with a micropipette. Three days after the CTB injection, mice were perfused with 4% PFA and optic nerves and retinas were collected. Fixed optic nerves containing injury sites were sectioned longitudinally (10 μm) and 5 representative sections were selected from each optic nerve by visualizing the most regenerating axons along the sections. We counted regenerating axons crossing several lines past the lesion and calculated their number by dividing the axon number by the nerve size at each distance. Following 2-hour post-fixation in the same PFA and overnight incubation in 30% sucrose, we immunostained the whole-mounted retina with an antibody for Tuj1 and examined the number of surviving RGCs in retina ipsilateral to injury. After quantifying the average number of Tuj1^+^ cells per field, we obtained the total number of viable RGCs by multiplying the figure by retinal area, as reported previously [13].

### RNA-seq data analysis

Analysis of RNA-seq data was performed as described previously [81]. Alignments of the sequencing data of three WT and three knockout samples were performed using STAR v2.5.2a to Ensembl *Drosophila* reference genome BDGP6.46 [82]. The multimapping and chimeric alignments were discarded, and only uniquely mapped reads were quantified at the gene level and summarized to gene counts using STAR-quantMode (GeneCounts). Differential gene expression analysis between WT and mutant samples was performed using DESeq2 (v1.36.0) in R (v3.6.0) after genes whose average counts were lower than the 10 were discarded [83]. Normalized gene counts of Rab10 and Piezo calculated by DESeq2 were used for visualization.

### Statistical analysis

The sample sizes are similar to those reported in previous publications and the statistical analyses were done afterward without interim data analysis. The values of “*n*” (sample size) are provided in the figure legends. All data were collected and processed randomly. Data are expressed as mean ± SEM in bar graphs, if not mentioned otherwise. No data points were excluded. Two-tailed unpaired Student’s *t*-test was performed for comparison between two groups of samples. One-way ANOVA followed by multiple comparison test was performed for comparisons among three or more groups of samples. Two-way ANOVA followed by multiple comparison test was performed for comparisons between two or more curves. Fisher’s exact test was used to compare the percentage, each group was compared with the first row of dataset. All the mutants and RNAis were compared to WT unless specified otherwise. Statistical significance was assigned, *p < 0.05, **p < 0.01, ***p < 0.001.

## Supporting information

S1 Fig Loss of Rtca doesn’t alter the glial scar size. Astrocyte scar border is marked by red dotted line and reactive astrocytes in spared but reactive neural tissue is outlined by yellow dotted line. Analyzed by unpaired t test, *p* = 0.8380, 0.4946, 0.8355, *n =* 5. Scale bar, 200 μm.
(EPS)

S2 Fig RtcB cooperates with its cofactor Archease to promote axon regeneration. (A) Generation of *RtcB*^*1-4B*^ mutant flies. Upper panel: proposed model for Rtca and RtcB regulating axon regeneration in opposite directions via competition to splice *Xbp1* mRNA. Middle panel: design of the gRNA targeting RtcB. Lower panel: CRISPR editing results in frameshift and truncates the protein. (B and C) *RtcB*^*1-4B*^ reduces axon regeneration. (B) C4da neuron axons were injured and regeneration was assessed at 48 h AI. Axons are highlighted by the dotted green line. The injury site is marked by the red dashed circle, regenerating and non-regenerating axons are labeled by arrowheads and arrow, respectively. Scale bar, 20 μm. (C) Quantification of axon regeneration by regeneration percentage (upper panel, Fisher’s exact test, *p* = 0.0341) and regeneration index (lower panel, unpaired t test, *p* = 0.0103). *n =* 36 and 54 neurons. (D and E) Compared with *RtcB*^*1-4B*^ or *Archease*^*PB*^ heterozygotes, axon regeneration is significantly decreased in *RtcB* and *Archease* transheterozygotes. (D) C4da axons were injured and regeneration was assessed at 48 h AI. Scale bar, 20 μm. (E) Quantification of axon regeneration percentage (upper panel, Fisher’s exact test) and regeneration index (lower panel, Kruskal-Wallis test followed by Dunn’s multiple comparisons test). *n =* 32, 27 and 47 neurons. *p < 0.05, **p < 0.01, ***p < 0.001. (F and G) Schematics showing the axon regeneration quantification for C4da and C3da neurons. Regeneration percentage accounts for the percent of injured axons that show obvious regeneration, which is defined by clear regeneration of the axon beyond the retracted axon stem. Regenerating axons (green) marked here with two red arrowheads Regeneration index is calculated by normalizing the axon length increase to the scaling length (the length of the magenta dashed line) which stretches from the base of the axon to the axon convergence point (blue arrowhead).
(EPS)

S3 Fig Overexpressing RtcB but not Archease is sufficient to increase regeneration. (A) C3da neuron axons were injured and assayed at 72 h AI. The injury site is marked by the dashed circle, regenerating axon is marked by arrowheads and non-regenerating axon is labeled by arrow. (B) Quantification of axon regeneration by regeneration percentage (upper panel, Fisher’s exact test, *p* > 0.9999, *p* = 0.0123) and regeneration index (lower panel, one-way ANOVA followed by Dunnett’s multiple comparisons test). *n =* 37, 18 and 36 neurons. Scale bar, 20 μm.
(EPS)

S4 Fig Comparing intracellular and cytoplasmic Piezo expression. GFP-Piezo intensity in the peripheral cell body, intracellularly and in the cytoplasm is quantified. The nucleus is marked by RedStinger. The GFP-Piezo peripheral/intracellular and peripheral/cytoplasm ratios are not statistically different. Analyzed by unpaired t test, *p* = 0.2495. Scale bar, 20 μm.
(EPS)

S5 Fig Piezo localization is not regulated by RtcB, and mys overexpression does not rescue Rtca mutant phenotypes. (A) GFP-Piezo localization to the peripheral cell body in C3da neurons is not altered by Archease LoF or RtcB overexpression. Rab10-CA overexpression increases overall GFP-Piezo expression in the soma and processes. The soma is marked by the blue dashed line, axon and dendrites are marked by arrows. Scale bar, 20 μm. (B-E) Quantification of GFP-Piezo peripheral/intracellular ratio, and its mean intensity normalized to mRFP, in (A). Analyzed by unpaired t test. *n* = 21, 19 (B), 20, 22 (C-E) neurons. (F) C3da neuron overexpression of mPiezo1 or mys fails to suppress the enhanced axon regeneration in Rtca knockout, as shown by regeneration percentage (Fisher’s exact test) and regeneration index (one-way ANOVA followed by Hold Sidak’s multiple comparisons test). *n* = 37, 23, 23, 25 neurons.(G) C3da neuron overexpression of mys does not rescue GFP-Piezo localization or increase soma intensity in *Rtca* knockout. Analyzed by unpaired t test. *n* = 22, 24 neurons. *p < 0.05, **p < 0.01, ***p < 0.001.
(EPS)

S6 Fig Rab10 knockdown rescues the impaired regeneration in Rtca overexpression neurons. (A) C4da neuron axons were injured and assayed at 48 h AI. The injury site is marked by the dashed circle, regenerating and non-regenerating axons are marked by arrowheads and arrow. (B) Quantification of axon regeneration by regeneration percentage (upper panel, Fisher’s exact test, *p* = 0.1783, 0.2517) and regeneration index (lower panel, unpaired *t*-test between *ppk-Gal4 > Rtca* and *ppk-Gal4 > Rtca; Rab10 RNAi*, *p* = 0.0401). *n* = 36, 38, 32 neurons. Scale bar, 20 μm. *p < 0.05.
(EPS)

S7 Fig Loss of Rab10 impairs the activation of Piezo’s downstream component and affects endosome trafficking. (A and B) Piezo’s downstream effector pCaMKII accumulated in 50% WT and 29.41% C3da neuron Rab10 knockdown axon tips at 48 h AI. The axon terminal is marked by the dashed circle. Analyzed Fisher’s exact test, *n* = 34, 34 neurons. Analyzed by Fisher’s exact test, *p* = 0.1364. Scale bar, 10 μm.(C-E) Live image showing GFP-Rab5 fluorescence (C) and GFP-Rab5 positive puncta area (E) are increased in Rab10 knockdown neurons at 24 h AI. The injury site is outlined by the dashed circles. Scale bar, 10 μm. (D) Unpaired *t*-test is used to analyze the difference between Ctrl and Rab10 knockdown C3da neurons at 8 h, 24 h, and 48 h AI. *n* = 36, 26, 30, 47, 29, 35, *p* = 0.179, 0.0265, 0.124. (E) To assess GFP-Rab5 puncta area, the neuron soma was outlined in ImageJ and Analyze Particles was used. Analyzed by unpaired *t*-test, *n* = 40, 46, *p* = 0.0153. (F) Piezo is detected in GFP-Rab5 positive puncta. *Piezo-Flag* knock-in fly stock was generated by fusing Flag to the C-terminal of Piezo. Larvae were dissected at 24 h AI, and their body walls were fixed and stained for GFP and Flag. Scale bar, 5 μm. *p < 0.05.
(EPS)

S8 Fig Independent of how many optical slices were analyzed, *Rab10*^-^ reduces Piezo’s localization to the peripheral cell body after injury. (A) Representative image stacks of *WT* and *Rab10*^-^ neurons that encompass the whole membrane of the cell body (5–6 slices), center of the cell body (2–3 slices) or a single optical plane (1 slice). (B) Analysis of *WT* and *Rab10*^-^ data in each of these conditions showed similar results. Scale bar, 5 μm. Analyzed by Mann-Whitney test. ***p < 0.001.
(EPS)

S9 Fig Localization of Piezo visualized in live larvae with the secreted split-GFP system. (A) Cartoon of the secreted-split-GFP-based approach to label Piezo when it is inserted into the neuronal cell membrane. GFP(11) is positioned on an extracellular loop of Piezo. Secreted-GFP(1–10) (secGFP(1–10)) is expressed by the fat bodies using DcG-Gal4, which secrete secGFP(1–10) into the hemolymph that circulates throughout larvae. Neither GFP(11) nor secGFP(1–10) is fluorescent on its own. When GFP(11) binds secGFP(1–10), resulting in reconstitution of GFP and fluorescent signal. (B) Representative images of GFP fluorescence in the dendrites, cell body, and axon of C3da (teal) and C1da (pink outline) neurons expressing Piezo tagged with 3 copies of GFP(11) on an extracellular (EC) loop (Piezo::GFP(11)x3, in WT, *Rtca* knockout and *Rab10* knockout. (C) Quantification of GFP peripheral/intracellular ratio for C3da neurons. Scale bar, 20 μm. Analyzed by one-way ANOVA followed by Dunnett’s multiple comparisons test. *n* = 10, 11, 10 neurons. ***p < 0.001.
(EPS)

S10 Fig mys is expressed by C3da neurons- (A) Immunostaining of endogenous mys using the anti-mys antibody shows reduced intensity (normalized to Tomato) in uninjured and injured C3da neurons after C3da neuron-specific mys knockdown. The soma is outlined. Analyzed by two-way ANOVA followed by Sidak’s multiple comparisons test. n = 9, 13, 14, 11 neurons.(B) Surface immunostaining of V5 using the non-permeabilized protocol in the *mys-V5* strain shows reduced intensity (normalized to Control) in uninjured C3da neurons after C3da neuron-specific mys knockdown. The soma is outlined. Scale bar, 20 μm. Analyzed by unpaired *t*-test, *n* = 15, 23 neurons. *p < 0.05, **p < 0.01, ***p < 0.001.
(EPS)

S11 Fig mys is important for Piezo targeting to the periphery of the soma. Piezo’s enrichment in the injured axon tip is attenuated in mys knockdown neurons at 24 h AI. The growth cone is highlighted by the dotted white circle. *n =* 51 and 40 neurons. Scale bar, 20 μm. Analyzed by Fisher’s exact test, *p* = 0.0172. *p < 0.05, ***p < 0.001.
(EPS)

S12 Fig Expressing mys attenuates the enhanced regeneration capacity in Rab10 knockdown neurons. (A) C3da neuron axons were injured and assayed at 72 h AI. The injury site is marked by the dashed circle, the regenerating axon is labeled by arrowheads and non-regenerated axon is marked by arrow. (B) Quantification of axon regeneration by regeneration percentage (upper panel, Fisher’s exact test, *p* = 0.2273, *p* = 0.0252) and regeneration index (lower panel, one-way ANOVA followed by Dunnett’s multiple comparisons test). *n =* 26, 46 and 40 neurons. Scale bar, 20 μm. *p < 0.05.
(EPS)

S13 Fig *Piezo1* conditional knockdown in RGCs promotes RGC survival and axon regeneration after optic nerve crush in adult mice. (A) Schematic of experimental protocols for the mice with optic nerve crush. AAV2-GFP or AAV2-Cre was injected into the retina intravitreally 7 days before injury. CTB was injected at 18 days after optic nerve crush and mice were perfused at 21 days after injury. (B-E) *Piezo1* conditional knockout (*Piezo1 cKO*) enhances RGC axon regrowth in the mice CNS. (B) Representative images of optic nerve after crush in control and *Piezo1 cKO* mice. Scale bar, 100 μm. (C) Representative images of the RGCs. Scale bar, 50 μm. (D) Quantification of the axon numbers extending beyond the injury cite. (E) Quantification of RGC survival rate after injury. *n =* 6 and 7 mice. Data are analyzed by two-way ANOVA (D) or unpaired *t*-test (E). **p < 0.01, ***p < 0.001.
(EPS)

S14 Fig Proposed genetic model of the Rtca pathway in mediating axon regeneration. (EPS)

S1 Data Primary data for all for the quantifications in each figure. (XLSX)

## References

[1] Mahar M, Cavalli V. Intrinsic mechanisms of neuronal axon regeneration. Nat Rev Neurosci. 2018;19(6):323–37. doi: 10.1038/s41583-018-0001-8 29666508 PMC5987780

[2] He Z, Jin Y. Intrinsic Control of Axon Regeneration. Neuron. 2016;90(3):437–51. doi: 10.1016/j.neuron.2016.04.022 27151637

[3] Duan X, Qiao M, Bei F, Kim I-J, He Z, Sanes JR. Subtype-specific regeneration of retinal ganglion cells following axotomy: effects of osteopontin and mTOR signaling. Neuron. 2015;85(6):1244–56. doi: 10.1016/j.neuron.2015.02.017 25754821 PMC4391013

[4] Chuang Y-C, Lee C-H, Sun W-H, Chen C-C. Involvement of advillin in somatosensory neuron subtype-specific axon regeneration and neuropathic pain. Proc Natl Acad Sci U S A. 2018;115(36):E8557–66. doi: 10.1073/pnas.1716470115 30126982 PMC6130359

[5] Zheng B, Tuszynski MH. Regulation of axonal regeneration after mammalian spinal cord injury. Nat Rev Mol Cell Biol. 2023;24(6):396–413. doi: 10.1038/s41580-022-00562-y 36604586

[6] Varadarajan SG, Hunyara JL, Hamilton NR, Kolodkin AL, Huberman AD. Central nervous system regeneration. Cell. 2022;185(1):77–94. doi: 10.1016/j.cell.2021.10.029 34995518 PMC10896592

[7] Song Y, Ori-McKenney KM, Zheng Y, Han C, Jan LY, Jan YN. Regeneration of Drosophila sensory neuron axons and dendrites is regulated by the Akt pathway involving Pten and microRNA bantam. Genes Dev. 2012;26(14):1612–25. doi: 10.1101/gad.193243.112 22759636 PMC3404388

[8] Song Y, Sretavan D, Salegio EA, Berg J, Huang X, Cheng T, et al. Regulation of axon regeneration by the RNA repair and splicing pathway. Nat Neurosci. 2015;18(6):817–25. doi: 10.1038/nn.4019 25961792 PMC4446171

[9] Vargas EJM, Matamoros AJ, Qiu J, Jan CH, Wang Q, Gorczyca D, et al. The microtubule regulator ringer functions downstream from the RNA repair/splicing pathway to promote axon regeneration. Genes Dev. 2020;34(3–4):194–208. doi: 10.1101/gad.331330.119 31919191 PMC7000917

[10] Li F, Lo TY, Miles L, Wang Q, Noristani HN, Li D, et al. The Atr-Chek1 pathway inhibits axon regeneration in response to Piezo-dependent mechanosensation. Nat Commun. 2021;12(1):3845. doi: 10.1038/s41467-021-24131-7 34158506 PMC8219705

[11] Song Y, Li D, Farrelly O, Miles L, Li F, Kim SE, et al. The Mechanosensitive Ion Channel Piezo Inhibits Axon Regeneration. Neuron. 2019;102(2):373-389.e6. doi: 10.1016/j.neuron.2019.01.050 30819546 PMC6487666

[12] Chen CC-H, Schweinsberg PJ, Vashist S, Mareiniss DP, Lambie EJ, Grant BD. RAB-10 is required for endocytic recycling in the Caenorhabditis elegans intestine. Mol Biol Cell. 2006;17(3):1286–97. doi: 10.1091/mbc.e05-08-0787 16394106 PMC1382317

[13] Nathan FM, Ohtake Y, Wang S, Jiang X, Sami A, Guo H, et al. Upregulating Lin28a Promotes Axon Regeneration in Adult Mice with Optic Nerve and Spinal Cord Injury. Mol Ther. 2020;28(8):1902–17. doi: 10.1016/j.ymthe.2020.04.010 32353321 PMC7403348

[14] Rosenzweig ES, McDonald JW. Rodent models for treatment of spinal cord injury: research trends and progress toward useful repair. Curr Opin Neurol. 2004;17(2):121–31. doi: 10.1097/00019052-200404000-00007 15021237

[15] Liu K, Lu Y, Lee JK, Samara R, Willenberg R, Sears-Kraxberger I, et al. PTEN deletion enhances the regenerative ability of adult corticospinal neurons. Nat Neurosci. 2010;13(9):1075–81. doi: 10.1038/nn.2603 20694004 PMC2928871

[16] Fu Q, Hue J, Li S. Nonsteroidal anti-inflammatory drugs promote axon regeneration via RhoA inhibition. J Neurosci. 2007;27(15):4154–64. doi: 10.1523/JNEUROSCI.4353-06.2007 17428993 PMC6672522

[17] Dill J, Wang H, Zhou F, Li S. Inactivation of glycogen synthase kinase 3 promotes axonal growth and recovery in the CNS. J Neurosci. 2008;28(36):8914–28. doi: 10.1523/JNEUROSCI.1178-08.2008 18768685 PMC6670875

[18] Lang BT, Cregg JM, DePaul MA, Tran AP, Xu K, Dyck SM, et al. Modulation of the proteoglycan receptor PTPσ promotes recovery after spinal cord injury. Nature. 2015;518(7539):404–8. doi: 10.1038/nature13974 25470046 PMC4336236

[19] Ohtake Y, Park D, Muneer PMA, Li H, Xu B, Sharma K, et al. The effect of systemic PTEN antagonist peptides on axon growth and functional recovery after spinal cord injury. Biomaterials. 2014;35(16):4610–26. doi: 10.1016/j.biomaterials.2014.02.037 24630093 PMC4195449

[20] Weidner N, Ner A, Salimi N, Tuszynski MH. Spontaneous corticospinal axonal plasticity and functional recovery after adult central nervous system injury. Proc Natl Acad Sci U S A. 2001;98(6):3513–8. doi: 10.1073/pnas.051626798 11248109 PMC30684

[21] Deumens R, Koopmans GC, Joosten EAJ. Regeneration of descending axon tracts after spinal cord injury. Prog Neurobiol. 2005;77(1–2):57–89. doi: 10.1016/j.pneurobio.2005.10.004 16271433

[22] Pearse DD, Pereira FC, Marcillo AE, Bates ML, Berrocal YA, Filbin MT, et al. cAMP and Schwann cells promote axonal growth and functional recovery after spinal cord injury. Nat Med. 2004;10(6):610–6. doi: 10.1038/nm1056 15156204

[23] Raineteau O, Schwab ME. Plasticity of motor systems after incomplete spinal cord injury. Nat Rev Neurosci. 2001;2(4):263–73. doi: 10.1038/35067570 11283749

[24] Blesch A, Tuszynski MH. Spinal cord injury: plasticity, regeneration and the challenge of translational drug development. Trends Neurosci. 2009;32(1):41–7. doi: 10.1016/j.tins.2008.09.008 18977039

[25] Steward O, Zheng B, Tessier-Lavigne M. False resurrections: distinguishing regenerated from spared axons in the injured central nervous system. J Comp Neurol. 2003;459(1):1–8. doi: 10.1002/cne.10593 12629662

[26] Unlu I, Lu Y, Wang X. The cyclic phosphodiesterase CNP and RNA cyclase RtcA fine-tune noncanonical XBP1 splicing during ER stress. J Biol Chem. 2018;293(50):19365–76. doi: 10.1074/jbc.RA118.004872 30355738 PMC6302167

[27] Jurkin J, Henkel T, Nielsen AF, Minnich M, Popow J, Kaufmann T, et al. The mammalian tRNA ligase complex mediates splicing of XBP1 mRNA and controls antibody secretion in plasma cells. EMBO J. 2014;33(24):2922–36. doi: 10.15252/embj.201490332 25378478 PMC4282640

[28] Kosmaczewski SG, Han SM, Han B, Irving Meyer B, Baig HS, Athar W, et al. RNA ligation in neurons by RtcB inhibits axon regeneration. Proc Natl Acad Sci U S A. 2015;112(27):8451–6. doi: 10.1073/pnas.1502948112 26100902 PMC4500288

[29] Kim SE, Coste B, Chadha A, Cook B, Patapoutian A. The role of Drosophila Piezo in mechanical nociception. Nature. 2012;483(7388):209–12. doi: 10.1038/nature10801 22343891 PMC3297676

[30] Coste B, Mathur J, Schmidt M, Earley TJ, Ranade S, Petrus MJ, et al. Piezo1 and Piezo2 are essential components of distinct mechanically activated cation channels. Science. 2010;330(6000):55–60. doi: 10.1126/science.1193270 20813920 PMC3062430

[31] Li K, Guo Y, Wang Y, Zhu R, Chen W, Cheng T, et al. Drosophila TMEM63 and mouse TMEM63A are lysosomal mechanosensory ion channels. Nat Cell Biol. 2024;26(3):393–403. doi: 10.1038/s41556-024-01353-7 38388853 PMC10940159

[32] Dunst S, Kazimiers T, von Zadow F, Jambor H, Sagner A, Brankatschk B, et al. Endogenously tagged rab proteins: a resource to study membrane trafficking in Drosophila. Dev Cell. 2015;33(3):351–65. doi: 10.1016/j.devcel.2015.03.022 25942626 PMC4431667

[33] Lerner DW, McCoy D, Isabella AJ, Mahowald AP, Gerlach GF, Chaudhry TA, et al. A Rab10-dependent mechanism for polarized basement membrane secretion during organ morphogenesis. Dev Cell. 2013;24(2):159–68. doi: 10.1016/j.devcel.2012.12.005 23369713 PMC3562474

[34] Chua CEL, Tang BL. Rab 10-a traffic controller in multiple cellular pathways and locations. J Cell Physiol. 2018;233(9):6483–94. doi: 10.1002/jcp.26503 29377137 PMC13482492

[35] Wang T, Liu Y, Xu X-H, Deng C-Y, Wu K-Y, Zhu J, et al. Lgl1 activation of rab10 promotes axonal membrane trafficking underlying neuronal polarization. Dev Cell. 2011;21(3):431–44. doi: 10.1016/j.devcel.2011.07.007 21856246

[36] Xu X-H, Deng C-Y, Liu Y, He M, Peng J, Wang T, et al. MARCKS regulates membrane targeting of Rab10 vesicles to promote axon development. Cell Res. 2014;24(5):576–94. doi: 10.1038/cr.2014.33 24662485 PMC4011341

[37] Lei M, Wang W, Zhang H, Gong J, Cai H, Wang Z, et al. Piezo1 Regulates Stiffness-Dependent DRG Axon Regeneration via Modifying Cytoskeletal Dynamics. Adv Sci (Weinh). 2024;11(47):e2405705. doi: 10.1002/advs.202405705 39514408 PMC11653623

[38] Mitchell JW, Midillioglu I, Schauer E, Wang B, Han C, Wildonger J. Coordination of Pickpocket ion channel delivery and dendrite growth in Drosophila sensory neurons. PLoS Genet. 2023;19(11):e1011025. doi: 10.1371/journal.pgen.1011025 37943859 PMC10662761

[39] Coste B, Murthy SE, Mathur J, Schmidt M, Mechioukhi Y, Delmas P, et al. Piezo1 ion channel pore properties are dictated by C-terminal region. Nat Commun. 2015;6:7223. doi: 10.1038/ncomms8223 26008989 PMC4445471

[40] Yao M, Tijore A, Cheng D, Li JV, Hariharan A, Martinac B, et al. Force- and cell state-dependent recruitment of Piezo1 drives focal adhesion dynamics and calcium entry. Sci Adv. 2022;8(45):eabo1461. doi: 10.1126/sciadv.abo1461 36351022 PMC9645726

[41] McHugh BJ, Buttery R, Lad Y, Banks S, Haslett C, Sethi T. Integrin activation by Fam38A uses a novel mechanism of R-Ras targeting to the endoplasmic reticulum. J Cell Sci. 2010;123(Pt 1):51–61. doi: 10.1242/jcs.056424 20016066 PMC2794710

[42] Howe EN, Burnette MD, Justice ME, Schnepp PM, Hedrick V, Clancy JW, et al. Rab11b-mediated integrin recycling promotes brain metastatic adaptation and outgrowth. Nat Commun. 2020;11(1):3017. doi: 10.1038/s41467-020-16832-2 32541798 PMC7295786

[43] Sarov M, Barz C, Jambor H, Hein MY, Schmied C, Suchold D, et al. A genome-wide resource for the analysis of protein localisation in Drosophila. Elife. 2016;5:e12068. doi: 10.7554/eLife.12068 26896675 PMC4805545

[44] Bunch TA, Salatino R, Engelsgjerd MC, Mukai L, West RF, Brower DL. Characterization of mutant alleles of myospheroid, the gene encoding the beta subunit of the Drosophila PS integrins. Genetics. 1992;132(2):519–28. doi: 10.1093/genetics/132.2.519 1427041 PMC1205153

[45] Wang X-W, Yang S-G, Zhang C, Hu M-W, Qian J, Ma J-J, et al. Knocking Out Non-muscle Myosin II in Retinal Ganglion Cells Promotes Long-Distance Optic Nerve Regeneration. Cell Rep. 2020;31(3):107537. doi: 10.1016/j.celrep.2020.107537 32320663 PMC7219759

[46] Cuajungco MP, Grimm C, Oshima K, D’hoedt D, Nilius B, Mensenkamp AR, et al. PACSINs bind to the TRPV4 cation channel. PACSIN 3 modulates the subcellular localization of TRPV4. J Biol Chem. 2006;281(27):18753–62. doi: 10.1074/jbc.M602452200 16627472

[47] Carrillo-Garcia J, Herrera-Fernández V, Serra SA, Rubio-Moscardo F, Vogel-Gonzalez M, Doñate-Macian P, et al. The mechanosensitive Piezo1 channel controls endosome trafficking for an efficient cytokinetic abscission. Sci Adv. 2021;7(44):eabi7785. doi: 10.1126/sciadv.abi7785 34714681 PMC8555900

[48] Kumar V, Alla SR, Krishnan KS, Ramaswami M. Syndapin is dispensable for synaptic vesicle endocytosis at the Drosophila larval neuromuscular junction. Mol Cell Neurosci. 2009;40(2):234–41. doi: 10.1016/j.mcn.2008.10.011 19059483 PMC2697329

[49] Venken KJT, Schulze KL, Haelterman NA, Pan H, He Y, Evans-Holm M, et al. MiMIC: a highly versatile transposon insertion resource for engineering Drosophila melanogaster genes. Nat Methods. 2011;8(9):737–43. doi: 10.1038/nmeth.1662 21985007 PMC3191940

[50] Dharmalingam E, Haeckel A, Pinyol R, Schwintzer L, Koch D, Kessels MM, et al. F-BAR proteins of the syndapin family shape the plasma membrane and are crucial for neuromorphogenesis. J Neurosci. 2009;29(42):13315–27. doi: 10.1523/JNEUROSCI.3973-09.2009 19846719 PMC6665186

[51] Graus-Porta D, Blaess S, Senften M, Littlewood-Evans A, Damsky C, Huang Z, et al. Beta1-class integrins regulate the development of laminae and folia in the cerebral and cerebellar cortex. Neuron. 2001;31(3):367–79. doi: 10.1016/s0896-6273(01)00374-9 11516395

[52] Trombley S, Powell J, Guttipatti P, Matamoros A, Lin X, O’Harrow T, et al. Glia instruct axon regeneration via a ternary modulation of neuronal calcium channels in Drosophila. Nat Commun. 2023;14(1):6490. doi: 10.1038/s41467-023-42306-2 37838791 PMC10576831

[53] Jacques-Fricke BT, Seow Y, Gottlieb PA, Sachs F, Gomez TM. Ca2+ influx through mechanosensitive channels inhibits neurite outgrowth in opposition to other influx pathways and release from intracellular stores. J Neurosci. 2006;26(21):5656–64. doi: 10.1523/JNEUROSCI.0675-06.2006 16723522 PMC6675278

[54] Oñate M, Catenaccio A, Martínez G, Armentano D, Parsons G, Kerr B, et al. Activation of the unfolded protein response promotes axonal regeneration after peripheral nerve injury. Sci Rep. 2016;6:21709. doi: 10.1038/srep21709 26906090 PMC4764858

[55] Zou W, Yadav S, DeVault L, Nung Jan Y, Sherwood DR. RAB-10-Dependent Membrane Transport Is Required for Dendrite Arborization. PLoS Genet. 2015;11(9):e1005484. doi: 10.1371/journal.pgen.1005484 26394140 PMC4578882

[56] Taylor CA, Yan J, Howell AS, Dong X, Shen K. RAB-10 Regulates Dendritic Branching by Balancing Dendritic Transport. PLoS Genet. 2015;11(12):e1005695. doi: 10.1371/journal.pgen.1005695 26633194 PMC4669152

[57] McColl BW, Graham DI, Weir CJ, White F, Horsburgh K. Endocytic pathway alterations in human hippocampus after global ischemia and the influence of APOE genotype. Am J Pathol. 2003;162(1):273–81. doi: 10.1016/S0002-9440(10)63818-7 12507910 PMC1851134

[58] Caswell PT, Vadrevu S, Norman JC. Integrins: masters and slaves of endocytic transport. Nat Rev Mol Cell Biol. 2009;10(12):843–53. doi: 10.1038/nrm2799 19904298

[59] Sahgal P, Alanko J, Icha J, Paatero I, Hamidi H, Arjonen A, et al. GGA2 and RAB13 promote activity-dependent β1-integrin recycling. J Cell Sci. 2019;132(11):jcs233387. doi: 10.1242/jcs.233387 31076515

[60] Margadant C, Sonnenberg A. Integrin-TGF-beta crosstalk in fibrosis, cancer and wound healing. EMBO Rep. 2010;11(2):97–105. doi: 10.1038/embor.2009.276 20075988 PMC2828749

[61] Myers JP, Santiago-Medina M, Gomez TM. Regulation of axonal outgrowth and pathfinding by integrin-ECM interactions. Dev Neurobiol. 2011;71(11):901–23. doi: 10.1002/dneu.20931 21714101 PMC3192254

[62] Andrews MR, Czvitkovich S, Dassie E, Vogelaar CF, Faissner A, Blits B, et al. Alpha9 integrin promotes neurite outgrowth on tenascin-C and enhances sensory axon regeneration. J Neurosci. 2009;29(17):5546–57. doi: 10.1523/JNEUROSCI.0759-09.2009 19403822 PMC6665849

[63] Werner A, Willem M, Jones LL, Kreutzberg GW, Mayer U, Raivich G. Impaired axonal regeneration in alpha7 integrin-deficient mice. J Neurosci. 2000;20(5):1822–30. doi: 10.1523/JNEUROSCI.20-05-01822.2000 10684883 PMC6772931

[64] Hara M, Kobayakawa K, Ohkawa Y, Kumamaru H, Yokota K, Saito T, et al. Interaction of reactive astrocytes with type I collagen induces astrocytic scar formation through the integrin-N-cadherin pathway after spinal cord injury. Nat Med. 2017;23(7):818–28. doi: 10.1038/nm.4354 28628111

[65] Kingston R, Amin D, Misra S, Gross JM, Kuwajima T. Serotonin transporter-mediated molecular axis regulates regional retinal ganglion cell vulnerability and axon regeneration after nerve injury. PLoS Genet. 2021;17(11):e1009885. doi: 10.1371/journal.pgen.1009885 34735454 PMC8594818

[66] Chen X, Wanggou S, Bodalia A, Zhu M, Dong W, Fan JJ, et al. A Feedforward Mechanism Mediated by Mechanosensitive Ion Channel PIEZO1 and Tissue Mechanics Promotes Glioma Aggression. Neuron. 2018;100(4):799-815.e7. doi: 10.1016/j.neuron.2018.09.046 30344046

[67] Cheng B, Wan W, Huang G, Li Y, Genin GM, Mofrad MRK, et al. Nanoscale integrin cluster dynamics controls cellular mechanosensing via FAKY397 phosphorylation. Sci Adv. 2020;6(10):eaax1909. doi: 10.1126/sciadv.aax1909 32181337 PMC7056303

[68] Xiang Y, Yuan Q, Vogt N, Looger LL, Jan LY, Jan YN. Light-avoidance-mediating photoreceptors tile the Drosophila larval body wall. Nature. 2010;468(7326):921–6. doi: 10.1038/nature09576 21068723 PMC3026603

[69] Awasaki T, Lai S-L, Ito K, Lee T. Organization and postembryonic development of glial cells in the adult central brain of Drosophila. J Neurosci. 2008;28(51):13742–53. doi: 10.1523/JNEUROSCI.4844-08.2008 19091965 PMC6671902

[70] Han C, Jan LY, Jan Y-N. Enhancer-driven membrane markers for analysis of nonautonomous mechanisms reveal neuron-glia interactions in Drosophila. Proc Natl Acad Sci U S A. 2011;108(23):9673–8. doi: 10.1073/pnas.1106386108 21606367 PMC3111288

[71] Petersen LK, Stowers RS. A Gateway MultiSite recombination cloning toolkit. PLoS One. 2011;6(9):e24531. doi: 10.1371/journal.pone.0024531 21931740 PMC3170369

[72] Potter CJ, Tasic B, Russler EV, Liang L, Luo L. The Q system: a repressible binary system for transgene expression, lineage tracing, and mosaic analysis. Cell. 2010;141(3):536–48. doi: 10.1016/j.cell.2010.02.025 20434990 PMC2883883

[73] Kohrs FE, Daumann I-M, Pavlovic B, Jin EJ, Kiral FR, Lin S-C, et al. Systematic functional analysis of rab GTPases reveals limits of neuronal robustness to environmental challenges in flies. Elife. 2021;10:e59594. doi: 10.7554/eLife.59594 33666175 PMC8016483

[74] Zhang J, Schulze KL, Hiesinger PR, Suyama K, Wang S, Fish M, et al. Thirty-one flavors of Drosophila rab proteins. Genetics. 2007;176(2):1307–22. doi: 10.1534/genetics.106.066761 17409086 PMC1894592

[75] Young PE, Richman AM, Ketchum AS, Kiehart DP. Morphogenesis in Drosophila requires nonmuscle myosin heavy chain function. Genes Dev. 1993;7(1):29–41. doi: 10.1101/gad.7.1.29 8422986

[76] Suh JM, Gao X, McKay J, McKay R, Salo Z, Graff JM. Hedgehog signaling plays a conserved role in inhibiting fat formation. Cell Metab. 2006;3(1):25–34. doi: 10.1016/j.cmet.2005.11.012 16399502

[77] Barolo S, Castro B, Posakony JW. New Drosophila transgenic reporters: insulated P-element vectors expressing fast-maturing RFP. Biotechniques. 2004;36(3):436–40, 442. doi: 10.2144/04363ST03 15038159

[78] Gratz SJ, Cummings AM, Nguyen JN, Hamm DC, Donohue LK, Harrison MM, et al. Genome engineering of Drosophila with the CRISPR RNA-guided Cas9 nuclease. Genetics. 2013;194(4):1029–35. doi: 10.1534/genetics.113.152710 23709638 PMC3730909

[79] Emoto K, Parrish JZ, Jan LY, Jan Y-N. The tumour suppressor Hippo acts with the NDR kinases in dendritic tiling and maintenance. Nature. 2006;443(7108):210–3. doi: 10.1038/nature05090 16906135

[80] Stone MC, Albertson RM, Chen L, Rolls MM. Dendrite injury triggers DLK-independent regeneration. Cell Rep. 2014;6(2):247–53. doi: 10.1016/j.celrep.2013.12.022 24412365 PMC3954604

[81] Zhang F, Yoon K, Zhang DY, Kim N-S, Ming G-L, Song H. Epitranscriptomic regulation of cortical neurogenesis via Mettl8-dependent mitochondrial tRNA m3C modification. Cell Stem Cell. 2023;30(3):300-311.e11. doi: 10.1016/j.stem.2023.01.007 36764294 PMC10031801

[82] Dobin A, Davis CA, Schlesinger F, Drenkow J, Zaleski C, Jha S, et al. STAR: ultrafast universal RNA-seq aligner. Bioinformatics. 2013;29(1):15–21. doi: 10.1093/bioinformatics/bts635 23104886 PMC3530905

[83] Love MI, Huber W, Anders S. Moderated estimation of fold change and dispersion for RNA-seq data with DESeq2. Genome Biol. 2014;15(12):550. doi: 10.1186/s13059-014-0550-8 25516281 PMC4302049

